# The globally widespread genus *Sulfurimonas*: versatile energy metabolisms and adaptations to redox clines

**DOI:** 10.3389/fmicb.2015.00989

**Published:** 2015-09-16

**Authors:** Yuchen Han, Mirjam Perner

**Affiliations:** Molecular Biology of Microbial Consortia, Biocenter Klein Flottbek, University of HamburgHamburg, Germany

**Keywords:** *Sulfurimonas*, versatile metabolism, sulfur metabolism, hydrogen metabolism, horizontal gene transfer

## Abstract

*Sulfurimonas* species are commonly isolated from sulfidic habitats and numerous 16S rRNA sequences related to *Sulfurimonas* species have been identified in chemically distinct environments, such as hydrothermal deep-sea vents, marine sediments, the ocean’s water column, and terrestrial habitats. In some of these habitats, *Sulfurimonas* have been demonstrated to play an important role in chemoautotrophic processes. *Sulfurimonas* species can grow with a variety of electron donors and acceptors, which may contribute to their widespread distribution. Multiple copies of one type of enzyme (e.g., sulfide:quinone reductases and hydrogenases) may play a pivotal role in *Sulfurimonas*’ flexibility to colonize disparate environments. Many of these genes appear to have been acquired through horizontal gene transfer which has promoted adaptations to the distinct habitats. Here we summarize *Sulfurimonas*’ versatile energy metabolisms and link their physiological properties to their global distribution.

## Introduction

The epsilonproteobacterial genus *Sulfurimonas* was first proposed by Inagaki et al. (2003). *Epsilonproteobacteria* were originally considered as human and animal pathogens and known representatives included *Campylobacter, Helicobacter*, and *Arcobacter* species (Nakagawa and Takaki, 2009). However, in the last decade more and more non-pathogenic *Epsilonproteobacteria* have been isolated from different types of environments (Nakagawa and Takaki, 2009). Of these non-pathogenic *Epsilonproteobacteria* a great deal are exclusively associated with hydrothermal vent environments, e.g., *Caminibacter, Nautilia, Hydrogenimonas, Thioreductor, Nitratiruptor, Nitratifractor*, and *Lebetimonas* (cf., Campbell et al., 2006), but other species like *Sulfuricurvum kujiense* have so far only been detected in terrestrial environments, e.g., an underground crude-oil storage cavity at Kuji in Iwate, Japan (Engel et al., 2004; Kodama and Watanabe, 2004). In contrast, members of *Sulfurospirillum, Sulfurovum*, and *Sulfurimonas* have been found in hydrothermal vents, other marine habitats and terrestrial provinces (cf. Supplementary Table S1, Campbell et al., 2006; Nakagawa and Takaki, 2009), demonstrating their widespread occurrence. We here will focus on species of the genus *Sulfurimonas*, their global distribution, their versatile energy metabolisms and their adaptive abilities which may have represented one of the key features for successfully colonizing various habitats in the course of evolution.

**FIGURE 1:**
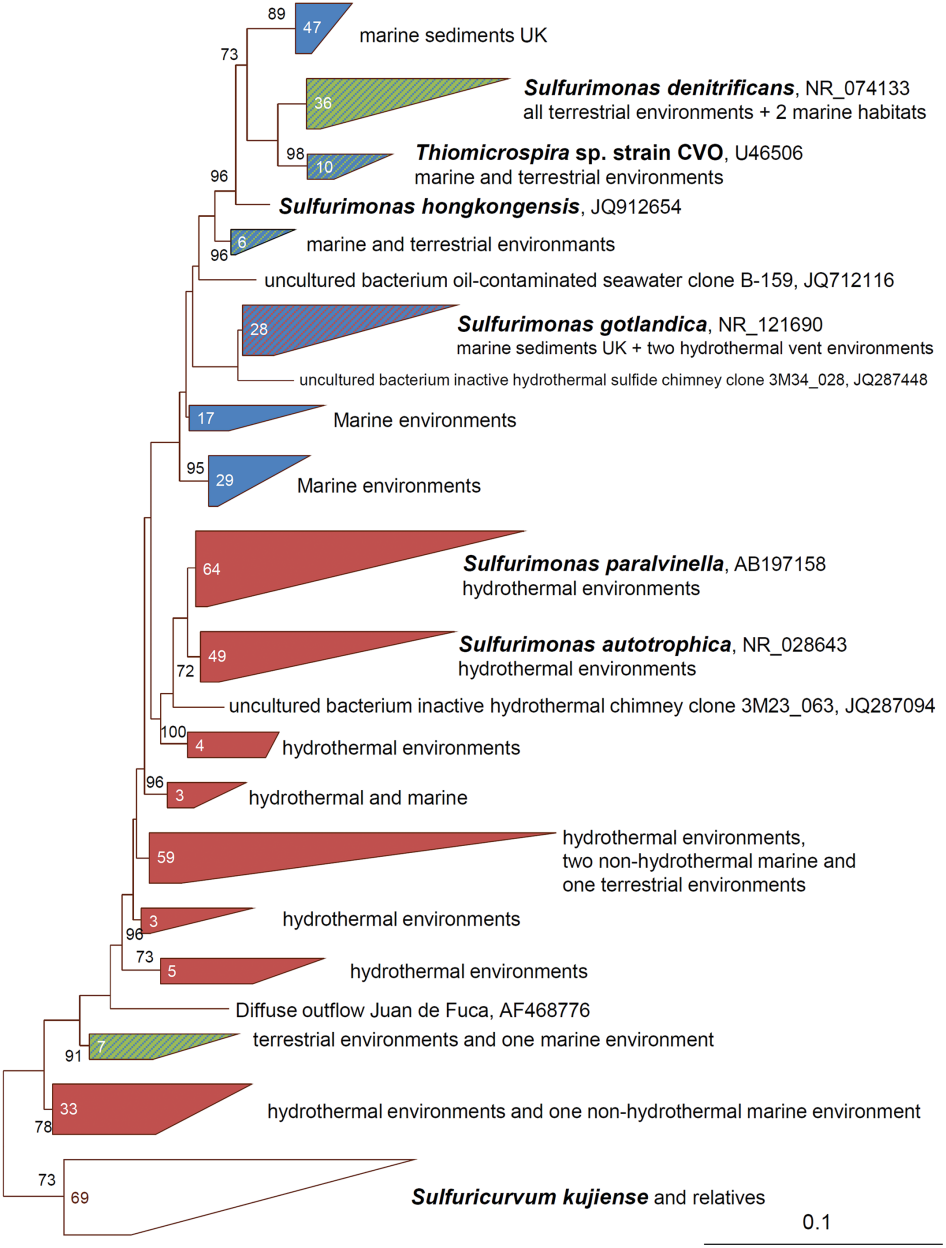
**Phylogenetic relationships of 16S rRNA gene sequences of *Sulfurimonas* species and closest relatives**. The 16S rRNA gene sequences were compiled by using the ARB software (www.arb-home.de) (Ludwig et al., 2004) and the alignments manually verified against known secondary structure regions. Maximum-Likelihood based trees with 1000 bootstrap replicates were constructed using PhyML (Guindon and Gascuel, 2003). Phylogenetic trees for 16S rRNA gene sequences were calculated considering only related sequences with lengths of at least 1400 bp. Trees were imported into ARB and shorter sequences were added subsequently to the trees without changing its topology. The number in the clade indicates the amount of sequences identified from the distinct environments. Only Bootstrap values ≥70 are shown. The scale bar represents the expected number of changes per nucleotide position.

Among the genus *Sulfurimonas* five species have been isolated and described: *Sulfurimonas denitrificans* (DSM 1251), previously named *Thiomicrospira denitrificans*, and *S. hongkongensis* strain AST-10^T^ (DSM 22096) were isolated from coastal marine sediments (Timmer-Ten Hoor, 1975; Takai et al., 2006; Cai et al., 2014), *S. autotrophica* strain OK10^T^ (DSM 16294) and *S. paralvinellae* strain GO25^T^ (DSM 17229) were isolated from sediments and polychaete nests in deep-sea hydrothermal vent fields (Inagaki et al., 2003; Takai et al., 2006), and *S. gotlandica* strain GD1^T^ (DSM 19862) was isolated from the pelagic redox cline in the central Baltic Sea (Grote et al., 2012; Labrenz et al., 2013). The genomes of *S. denitrificans, S. autotrophica*, and *S. gotlandica* have already been sequenced and genome sequencing of *S. hongkongensis* is currently in progress (Sievert et al., 2008b; Sikorski et al., 2010b; Grote et al., 2012; Cai et al., 2014). Based on 16S rRNA genes, a further species is phylogenetically placed within the *Sulfurimonas* group, namely *Thiomicrospira* sp. CVO (**Figure 1**). When *Thiomicrospira* sp. CVO was isolated from an oil field in Canada (Voordouw et al., 1996), the highest 16S rRNA gene similarity to an isolated strain was to that of *Thiomicrospira denitrificans* (96.1% similarity) and thus it was classified into the *Thiomicrospira* genus (Gevertz et al., 2000). However, *Thiomicrospira denitrificans* had been wrongly classified into the *Thiomicrospira* genus (*Gammaproteobacteria*) and was soon after reclassified into the *Sulfurimonas* genus (*Epsilonproteobacteria*) (Takai et al., 2006). Consequently, since *Thiomicrospira* sp. CVO phylogenetically groups among *Sulfurimonas* species (**Figure 1**), a reclassification of this strain into the *Sulfurimonas* genus needs to be discussed and we here incorporate it into our discussion on *Sulfurimonas* species.

The top 100 hits of Blast searches with 16S rRNA gene sequences of the cultured *Sulfurimonas* species demonstrate the widespread occurrence of affiliates of this genus (**Figure 1**). Sequences have been recovered from various marine environments such as hydrothermal vent habitats, the pelagic redox cline of the Baltic and Black Sea and coastal sediments but also terrestrial habitats like oil caves, contaminated ground water or sediments and sulfidic springs or supra-glacial spring systems (**Figure 1**, Voordouw et al., 1996; Watanabe et al., 2000; Kodama and Watanabe, 2003; Grote et al., 2008; Glaubitz et al., 2009; Yagi et al., 2009; Gleeson et al., 2011; Headd and Summers Engel, 2014). Besides being ubiquitously found across the globe, *Sulfurimonas* species can also make up large proportions of the bacterial community. For example, in the pelagic redox-cline of the central Baltic Sea they made up 30% of all cells and in plume waters and diffuse fluids of a deep-sea vent they comprised 70 and 20%, respectively, of the bacterial phylotype abundance (Grote et al., 2008; Akerman et al., 2013; Perner et al., 2013a). In case of the redox-cline of the Baltic Sea, the Black Sea and the plume waters, *Sulfurimonas* appears to be responsible for much of the chemoautotrophic activity as measured through incorporation of radioactively labeled bicarbonate (Grote et al., 2008; Glaubitz et al., 2009; Perner et al., 2013b) demonstrating its importance for microbial element cycling in some habitats. *Sulfurimonas*’ success has been attributed to its ability to grow chemolithoautotrophically, its metabolic versatility (use of various electron donors, electron acceptors, and inorganic carbon sources), its oxygen tolerance and its environmental sensing systems (Campbell et al., 2006; Sievert et al., 2008b; Grote et al., 2012; Perner et al., 2013a). We discuss possible horizontal gene transfer events for distinct key genes and link the ability of *Sulfurimonas* species to operate numerous energy metabolisms under various chemical conditions in different types of environments.

## Energy Metabolisms Among *Sulfurimonas* Species

### Sulfur Metabolism

The lineage *Sulfurimonas* combines a group of sulfur-oxidizing bacteria (Inagaki et al., 2003). Many kinds of reduced sulfur compounds, such as sulfide, elemental sulfur, thiosulfate and sulfite, can serve as an electron donor for the growth of *Sulfurimonas* species, but not all *Sulfurimonas* species can use all above mentioned sulfur compounds (**Table 1**). Besides using sulfur compounds for energy generation, they are also essential for biosynthesis, e.g., amino acids like cysteine, and thus need to be assimilated by the cells.

**Table 1:** List of electron donors, electron acceptors, and carbon sources for *Sulfurimonas* species.

	*Sulfurimonas denitrificans*	*S. hongkongensis*	*S. autotrophica*	*S. paralvinellae*	*S. gotlandica*	*Thiomicrospira* sp. CVO^1^
**Isolated source**	Coastal marine sediments		Deep-sea hydrothermal sediments	Nest of hydrothermal vent polychaetes	Marine sulfidic water of a pelagic redox zone	Terrestrial oil field
	Dutch Wadden Sea	Victoria Harbour, Hong Kong	Mid-Okinawa Trough, Japan		Central Baltic Sea	Saskatchewan, Canada
**Electron donors**						
Reduced inorganic sulfur compounds						
Sulfide	**+**	**+**	**+**	**-**	**+**	**+**
Element sulfur	**+**	NR	**+**	**+**	**+**	**+**
Thiosulfate	**+**	**+**	**+**	**+**	**+**	**-**
Sulfite	**-**	NR	**-**	**-**	**+**	NR
Hydrogen	**+**	**+**	**-**^2^	**+**	**+**	**-**
**Electron acceptors**						
Nitrate	**+**	**+**	**-**^2^	**+**	**+**	**+**
Nitrite	**+**	**-**	**-**	**-**	**+**	**+**
Oxygen	**+**	NR	**+**	**+**	**+**^2,3^	**+**
**Carbon source**						
Carbon dioxide/Bicarbonate	**+**	**+**	**+**	**+**	**+**	**+**
Acetate	NR	**-**	**-**	**-**	**+**	**+**
**Organic compounds**	Formate, fumarate, yeast extract, alcohol mix as electron donors	**-**	**-**	yeast extract as sulfur source	Formate, acetate, yeast extract, pyruvate, amino acid mix as electron donors	Acetate as carbon source
**Reference**	Timmer-Ten Hoor, 1975; Timmer-Ten Hoor, 1981; Labrenz et al., 2013	Cai et al., 2014	Inagaki et al., 2003	Takai et al., 2006	Grote et al., 2012; Labrenz et al., 2013	Voordouw et al., 1996; Gevertz et al., 2000; Kodama and Watanabe, 2003

*+, can be used; **-**, cannot be used; NR, not reported. ^1^*Thiomicrospira* sp. CVO is reclassified as a *Sulfurimonas* species in this review. ^2^The properties listed in this table were characterized by growth experiments. Because of the limitation of some experimental designs, more experiments are needed, e.g., growth experiments with more nutrients (For details, see the appropriate paragraph in the text). ^3^The growth of *S. gotlandica* with oxygen as an electron acceptor was not detected with growth experiments. However, the authors posited that *S. gotlandica* might be able to use oxygen as an electron acceptor and that the growth rate is merely too low to be detected with their experimental setup*.

#### Sulfide:Quinone Reductase

**FIGURE 2:**
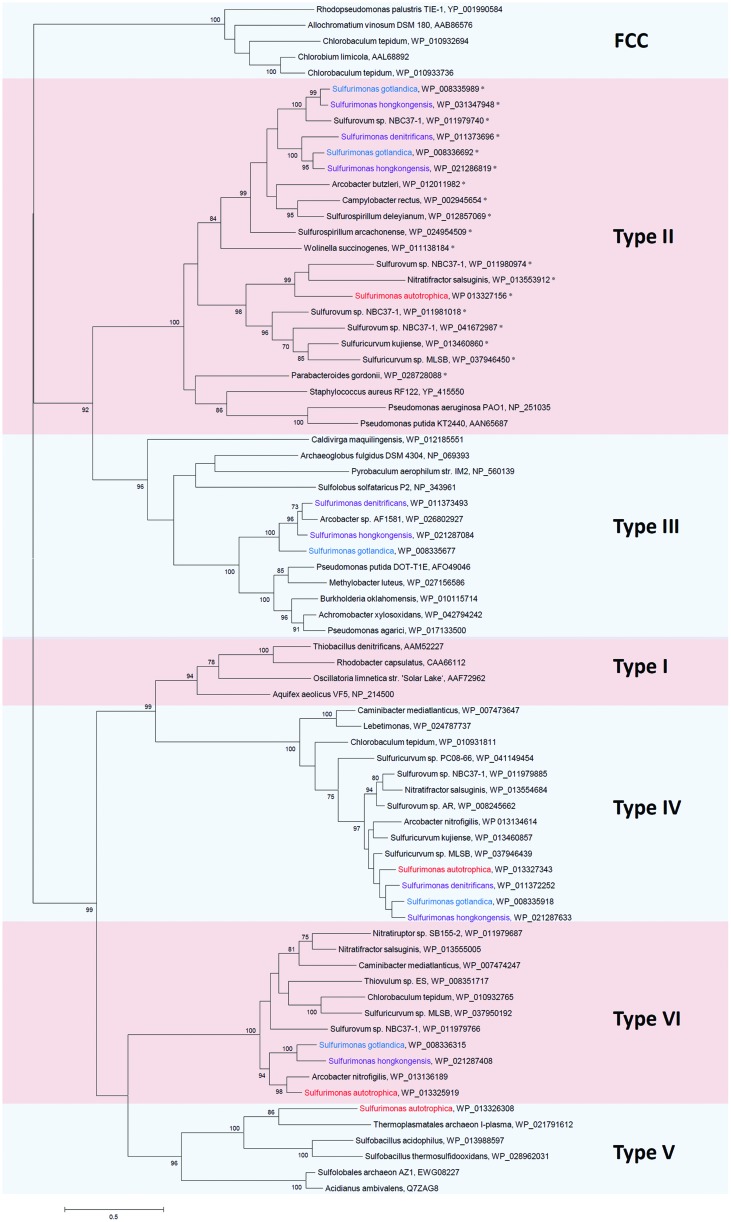
**Phylogenetic relationships of SQR sequences to those from *Sulfurimonas* species**. The sequences of SQRs were compared with the non-redundant protein database of NCBI by using BLASTP. The phylogenetic tree was constructed with MEGA6 (Tamura et al., 2013) based on the Maximum-likelihood method with 1000 bootstrap replications after multiple alignments with ClustalW (Larkin et al., 2007). The percentage of bootstrap resamplings ≥70 is indicated on the branches. The scale bar represents the expected number of changes per amino acids position. The types of SQRs are classified according to Marcia et al. (2010). Isolation sources of *Sulfurimonas* species are indicated in different colors: blue, marine non-vent water system; purple, marine sediments; red, hydrothermal environments. The proteins which has a TAT motif at N-terminus are marked with ‘^∗^’.

Except for *S. paralvinellae*, all strains can oxidize sulfide and produce sulfate as an end product and elemental sulfur and polysulfide as intermediate products. For chemolithotrophic bacteria two sulfide oxidizing pathways exist: in one pathway sulfide:quinone reductase (SQR, EC 1.8.5.4) and in the other pathway flavocytochrome *c* (FCC, also known as flavocytochrome *c* sulfide dehydrogenase) catalyzes the reaction (Griesbeck et al., 2000). The released electrons are donated to the electron transport chain either at the level of quinone, if SQR is used, or at the level of cytochrome *c*, if FCC is used (Griesbeck et al., 2000). Since no FCC encoding genes are found in any of the known *Sulfurimonas*’ genomes, sulfide oxidation is likely catalyzed by SQR. According to the structure-based sequence fingerprints, SQR proteins have been classified into six types, SQR Types I – VI (**Figure 2**, Pham et al., 2008; Marcia et al., 2010). However, several aspects on SQR functioning remain unresolved, including whether all Types of SQR bind quinones in the same way, and whether the different SQRs generate the same intermediates and same sulfur products during sulfide oxidation (Marcia et al., 2010).

Most of the genome sequenced *Epsilonproteobacteria* have Type II, IV, and VI and none have Type I (see Supplementary Table S1 for details). Interestingly, *Sulfurimonas* species appear to be the only *Epsilonproteobacteria* that have Type III (except for *Arcobacter* sp. AF1581) and Type V, aside from Type II, IV, and VI SQRs. Since Type IV SQR is so conserved among all sequenced *Sulfurimonas* species, it appears highly likely that it is vital for *Sulfurimonas*’ basic cell maintenance. One well characterized Type IV SQR is CT0117 (Accession No. WP_010931811) from *Chlorobium tepidum*. It is expressed constitutively but apparently primarily supports growth at sulfide levels between 2 and 4 mM (Chan et al., 2009). *C. tepidum* also contains a Type VI SQR (CT1087, Accession No. WP_010932765). Its transcript is induced at high sulfide concentrations and it is posited to be important for *C. tepidum*’s growth at sulfide concentrations above 4 mM (Chan et al., 2009). *S. denitrificans* is the only genome sequenced *Sulfurimonas* that lacks the Type VI SQR (**Figure 2**; Supplementary Table S1). Since the *Sulfurimonas* Type VI SQR phylogeny does not indicate multiple acquisitions from foreign hosts, but suggests that a common ancestor may have had a Type VI SQR, *S. denitrificans* appears to have lost its version in the course of time. The free sulfide concentration in tidal flats of the Dutch Wadden Sea, where *S. denitrificans* was originally isolated from, is much lower than 4 mM (on average <2.9 μM to maximal 21 μM; at two individual sites maximum sulfide concentrations of 587 μM and 1.8 mM were measured; Groenendaal, 1979). Other studies from the German Wadden Sea have yielded similar results (Jansen et al., 2009). Much of the sulfide appears to be buried in form of iron-sulfide (0.16–14 mM; Groenendaal, 1979). Thus, given that in *C. tepidum* the Type VI SQR is specialized to function at considerably higher sulfide concentrations, i.e., above 4 mM, than the Type IV, we hypothesize that *S. denitrificans* does not need a Type VI SQR because it would unlikely encounter free sulfide in the range of what the Type VI SQR from *C. tepidum* appears to be specialized on. *S. denitrificans* may also simply deal with elevated sulfide concentrations differently to *C. tepidum* and thus not need a SQR VI.

No Type I SQR was identified on any of the sequenced *Sulfurimonas* genomes. However, Type I and Type IV SQRs form a monophyletic clade (**Figure 2**), where sequences share conserved cysteines required for FAD binding and redox active coupling as well as conserved residues responsible for sulfide access (Pham et al., 2008; Marcia et al., 2009, 2010). This suggests that these two types of SQR may function in the same way and thus it is not surprising that *Sulfurimonas* species and as such other *Epsilonproteobacteria* only encode one of these two types.

The Type II SQR appears to have only low (millimolar) affinity to sulfide (cf. Marcia et al., 2010). It is the only SQR that can be found in multiple copies on various epsilonproteobacterial genomes (**Figure 2**, Supplementary Table S1). *Sulfurovum* sp. NBC 37-1 even has four copies (Nakagawa et al., 2007). *S. hongkongensis* and *S. gotlandica* have two copies each, which group together and which, in one case cluster together with the respective SQR from *S. denitrificans* (**Figure 2**). In contrast, *S. autotrophica*’s Type II SQR is phylogenetically distinct from the other *Sulfurimonas.* Distinct roles have been assigned to the Type II SQR: it is involved in sulfur assimilation in the non-pathogenic bacterium *Pseudomonas putida* KT2440 (Shibata and Kobayashi, 2006), in the heavy metal tolerance in the yeast *Saccharomyces pombe* (Vande Weghe and Ow, 1999) and in sulfide signaling in mammalian cells (Shahak and Hauska, 2008). Given that some *Sulfurimonas* species can considerably influence the mobility of Zn, Cu, and Pb through sulfide-oxidizing denitrification in contaminated marine sediments (Shao et al., 2009), some Type II SQRs from *Sulfurimonas* species may play a role in heavy metal tolerance. Interestingly, all Type II SQRs from *Sulfurimonas* species and non-*Sulfurimonas Epsilonproteobacteria* in Supplementary Table S1 have a two-arginine protein translocation (TAT) motif at the N-terminus (indicated in **Figure 2**). Protein folding in the periplasm may cause problems under specific environmental conditions, such as high salt concentrations (Palmer and Berks, 2012). Under conditions as found at highly reduced and acidic hydrothermal vents, toxic sulfidic environments or heavy metal-contaminated fields protein folding may also be impaired in the periplasm. Hence, in such a case it is useful if the protein can be folded and matured under more controlled conditions in the cytoplasm and then transported to the periplasmic side of the membrane via the translocation pathway (Palmer and Berks, 2012). The TAT motifs in Type II SQRs might be helpful for the translocation of folded and maturated Type II SQRs from the cytoplasm to the periplasmic side in *Sulfurimonas* and in other *Epsilonproteobacteria*.

Among the genome sequenced *Sulfurimonas* species only *S. autotrophica* contains a Type V SQR (Sikorski et al., 2010b). This *sqr* lineage is predominated by SQRs from thermoacidophilic bacteria and archaea (**Figure 2**). While some of these thermoacidophilic microorganisms have been isolated from deep-sea hydrothermal vents (*Sulfobacillus acidophilus*; Li et al., 2011), the genome sequences of other thermoacidophilic microorganisms with an SQR Type V closely related to that of *S. autotrophica* (**Figure 2**), originate from organisms that were isolated from acid mines (Golovacheva and Karavaiko, 1977; *Thermoplasmatales* archaeon I-plasma, Bioproject: PRJNA209804). These lineages, however, include species that have also been detected in deep-sea hydrothermal vents but for which currently no genome sequences are available (Reysenbach et al., 2006; Henneberger, 2008). *S. autotrophica* was isolated from a hydrothermal vent and thus is likely the only genome sequenced *Sulfurimonas* species that directly comes into contact with high abundances of thermoacidophiles. This may explain why it is the only *Sulfurimonas* we are aware of that appears to have acquired the Type V SQR. However, if acquiring the Type V SQR is of such an advantage, it does not explain why none of the other hydrothermal vent *Epsilonproteobacteria* carries a Type V SQR on their genome. For example, *S.* sp. NBC 37-1 has a very similar metabolism to *S. autotrophica* (both are mesophiles that can oxidize various reduced sulfur compounds, can use oxygen as electron acceptor and fix CO_2_ autotrophically) (Campbell et al., 2006), but *S.* sp. NBC 37-1 lacks a Type V SQR (Nakagawa et al., 2007). However, there is also a metabolic difference between these two species: *S*. sp. NBC-37 can additionally use hydrogen as an electron donor and nitrate as an electron acceptor, whereas *S. autotrophica* cannot (Inagaki et al., 2003; Nakagawa et al., 2007). Therefore, oxidation of reduced sulfur compounds coupled with oxygen as an electron acceptor is very crucial for *S. autotrophica*. Compared to Type I SQR from *Aquifex aeolicus*, the Type V SQR has a different ‘capping loop,’ which guarantees sulfide access to the oxidation site and it is expected that sulfide reaches the active site of the enzyme through a differently structured channel (Marcia et al., 2010). We posit that such a structure in Type V SQR may play an important role for sulfide oxidation to enhance energy generation in *S. autotrophica*. In the thermoacidophilic archaeon *Acidianus ambivalens* this type of SQR exhibits the highest activity at 70°C and pH 6.5 and only 3% activity was detected at 25°C and pH 6.5 (Brito et al., 2009). This indicates that Type V may have a tolerance to elevated temperatures. Since *S. autotrophica*’s gene clusters with archaeal *sqr* genes, it has likely acquired its Type V *sqr* gene from a member of the domain archaea through horizontal gene transfer.

*S. denitrificans, S. gotlandica*, and *S. hongkongensis* all contain a Type III SQR, which form a monophyletic group together with *Arcobacter* sp. AF1581 (**Figure 2**). Although, many Type III SQRs are associated with thermophilic archaea, the *Sulfurimonas*–*Arcobacter* clade group together with β- and γ-proteobacterial SQRs (**Figure 2**). Hence, it is unlikely that these SQRs are specialized to function at elevated temperatures, since several bacteria colonizing moderate temperature regimes have this SQR as well. The Type III SQRs are phylogenetically clustered with Type II SQRs, indicating that they may have similar functions. However, hardly anything is known about the bacterial Type III SQRs. Although a Type III *sqr*-gene (CT1025, accession No. NP_661917) has been identified in *C. tepidum*, no SQR activity was detected in the respective membrane proteins when both Type IV and VI *sqr* genes (CT0117 and CT1087) were deleted (Chan et al., 2009). Consequently, it can be assumed that under the provided conditions the Type III SQR is not catalyzing sulfide oxidation. It may, however, have a specific function under distinct environmental conditions. Since most *Epsilonproteobacteria* have one or more copies of Type II SQRs, Type III SQRs might not be crucial for *Epsilonproteobacteria*, which could explain why Type III SQRs are present in only very few epsilonproteobacterial species.

#### SOX Enzyme System

Oxidation of reduced sulfur compounds can also be catalyzed by the Sox pathway, which is a thiosulfate-oxidizing multi-enzyme system and a widespread mechanism within sulfur-oxidizing bacteria (Friedrich et al., 2005). SoxXA, SoxYZ, and SoxB make up the Sox multi-enzyme complex and can oxidize the reduced sulfur compounds stepwise, finally producing sulfate excreted out of cells (Frigaard and Dahl, 2008). In the Sox-mediated pathway SoxB encodes the key enzyme sulfate thiohydrolase. Except for *Thiomicrospira* sp. CVO, all *Sulfurimonas* species have been shown to utilize thiosulfate (**Table 1**). More so, all sequenced *Sulfurimonas* genomes encode all *sox* genes (*soxXYZAB*) required for a fully functional Sox multi-enzyme system. (Sievert et al., 2008b; Sikorski et al., 2010b; Grote et al., 2012). According to the presence of SoxCD, the Sox pathway can be grouped into two different versions: (1) SoxCD acts as a sulfur dehydrogenase, and no sulfur globule is accumulated or (2) sulfur is formed as an intermediate because of lacking SoxCD (Frigaard and Dahl, 2008). The *soxCD* genes were found on all of the sequenced *Sulfurimonas* genomes, indicating that these species perform the first Sox pathway with the presence of SoxCD as sulfur dehydrogenase, which oxidizes sulfane bound to the cysteine residue of SoxYZ to a sulfite anion. The key enzyme SoxB then catalyzes this sulfite anion to sulfate and releases a sulfate group from SoxYZ (Frigaard and Dahl, 2008). The phylogenetic tree of SoxB showed that the SoxB proteins from *Sulfurimonas* species are closely related and form a monophyletic group (Supplementary Figure S1A), consistent with previous studies (Hügler et al., 2011), and suggesting that it is an essential part of the *Sulfurimonas* metabolism. Sox enzyme systems are also found in several other *Epsilonproteobacteria* inhabiting different environments. They include *Sulfurovum, Sulfuricurvum, Nitratiruptor, Acrobacter*, and *Nitratifractor* (see Supplementary Table S1), although no growth of strain *Nitratifractor salsuginis* on thiosulfate could be shown (Nakagawa et al., 2005c).

#### Sulfite Dehydrogenase

Sulfite can be oxidized by the sulfite dehydrogenase (SorAB, EC 1.8.2.1), which is a soluble SOR and contains molybdenum pterin as a cofactor (Kappler and Dahl, 2001). SorAB directly oxidizes sulfite to sulfate with cytochrome *c* as an electron acceptor. Only few of the *Epsilonproteobacteria* genera have the SOR enzymes encoded on their genomes: besides *Sulfurimonas* this includes species of the *Sulfurovum* and *Sulfuricurvum* (Supplementary Table S1), which have been isolated from sulfide-rich environments (Nakagawa et al., 2005a; Hubert et al., 2012). *S. denitrificans* is the only genome sequenced *Sulfurimonas* species that does not encode for the *sorAB*-homologous genes, which coincides with its inability to grow on sulfite (Supplementary Figure S1B; **Table 1**, Timmer-Ten Hoor, 1981; Sievert et al., 2008b). Growth experiments showed that sulfite can indeed serve as an electron donor for the growth of *S. gotlandica* (all growth experiments are summarized in **Table 1**, Grote et al., 2012; Labrenz et al., 2013). However, no sulfite-based growth could be reported for *S. autotrophica* (Inagaki et al., 2003), despite SOR activity was detected in its cell extracts (Takai et al., 2005). Likewise, *S. paralvinellae*’s cell extract also exhibited SOR activity, but this species does not appear to use sulfite as an electron donor either (Takai et al., 2005, 2006). This, however, can be explained: unlike other sulfite oxidases (EC 1.8.3.1), pathways depending on SorAB are unable to use oxygen as an electron acceptor (Kappler and Dahl, 2001) and its cofactor molybdenum pterin is labile in the presence of oxygen (Rajagopalan and Johnson, 1992). Since the only electron acceptor that *S. autotrophica* can use is oxygen, it has always been cultivated in the presence of oxygen (Inagaki et al., 2003). Under this condition, however, SorAB from *S. autotrophica* might not be active because of its oxygen-sensitive cofactor. This is in line with its inability to use sulfite as an electron donor (**Table 1**). The SOR activities that were measured for *S. autotrophica* and *S. paralvinellae* may in fact reflect the activity of SoxCD in the cell extracts (Sievert et al., 2008a). It is homologous to SOR and has been demonstrated to act as a periplasmic SOR in the *Alphaproteobacterium Paracoccus pantotrophus* (Kappler and Dahl, 2001). In summary, we conclude that *S. gotlandica* expresses an active SOR, *S. autotrophica* contains an inactive SOR, likely because of its aerobic growth, and *sor* genes are completely lost in *S. denitrificans*. Since sulfide concentrations can be very low in the Dutch Wadden Sea sediments (Groenendaal, 1979), reactive and instable sulfite is likely very low as well. *S. denitrificans* may have lost these genes due to very low sulfite concentrations in the Dutch Wadden Sea habitat. Should sulfite accumulate intracellularly, the sox enzyme system could step in for detoxification of sulfite. The ability to use sulfite has not been examined in *S. hongkongensis*. Yet, given that both SorAs form a monophyletic cluster and appear to be similar in structure (Supplementary Figure S1B) we argue that *S. hongkongensis* may express an active SOR like *S. gotlandica*.

#### ATPS and APSR

Besides the above mentioned sulfur metabolisms, another two sulfur metabolizing pathways for either assimilatory sulfate reduction or dissimilatory sulfide oxidation exist that involve the intermediates adenylylsulfate (APS, also called adenosine-5′-phosphosulfate) or phosphoadenosine-5′-phosphosulfate (PAPS) and different types of APS reductase (APSR, EC 1.8.4.8 in assimilatory pathway and EC 1.8.99.2 in dissimilatory pathway) and ATP sulfurylase (ATPS, ATP: sulfate adenylyltransferase, EC 2.7.7.4) enzymes (Neumann et al., 2000; Kappler and Dahl, 2001; Frigaard and Dahl, 2008): in the assimilatory sulfate reduction pathway, sulfate is reduced to APS via the ATPS (two subunits, also called CysDN-type ATPS) and then to sulfite via the assimilatory APSR (one subunit, encoded by *cysH* gene). Alternatively, APS can also be phosphorylated to PAPS via the APS kinase (CysC) and then to sulfite via the PAPS reductase (also encoded by *cysH* gene; Neumann et al., 2000; Frigaard and Dahl, 2008). The sulfite is then reduced to sulfide via the sulfite reductase (encoded by *cysI*) (Neumann et al., 2000). The homologs of the CysDN-type ATPS were found in *S. denitrificans, S. gotlandica*, and *S. autotrophica* (cf. Supplementary Figure S1C) (Sievert et al., 2008b; Sikorski et al., 2010b; Grote et al., 2012) but appear to be missing in *S. hongkongensis*. The homologs of the CysH-type APSR (or PAPS reductase) and of CysI were found only in *S. denitrificans* and *S. gotlandica* (cf. Supplementary Figures S1D,E) (Sievert et al., 2008b; Grote et al., 2012). Additionally, CysC was found in *S. gotlandica* and *S. autotrophica* (cf. Supplementary Figure S1F). Hence, *S. denitrificans* is likely performing assimilatory sulfate reduction without PAPS as an intermediate, which was also proposed by Sievert et al. (2008b); and *S. gotlandica* is probably able to perform assimilatory sulfate reduction without and with PAPS as an intermediate. In contrast, *S. autotrophica* and *S. hongkongensis* are missing genes encoding key enzymes of this pathway and are thus likely either taking up the required sulfur molecules directly from the environment or have other means to produce the necessary sulfur compounds. In the dissimilatory sulfide oxidation pathway, sulfite is oxidized through the indirect and AMP-dependent oxidation by the dissimilatory APSR (two subunits, encoded by *aprBA*) to APS and then to sulfate via the ATPS (one subunit, also called as Sat-type ATPS). Interestingly enough, *S. denitrificans, S. gotlandica, S. autotrophica*, and *S. hongkongensis* all have a Sat-type ATPS (cf. Supplementary Figure S1C) (Sievert et al., 2008b; Sikorski et al., 2010b; Grote et al., 2012; Cai et al., 2014). However, since none of these species have an AprBA-type dissimilatory APSR, it appears highly unlikely that this Sat-type ATPS is involved in dissimilatory sulfide oxidation. Also, respective AprBA activities were not detected in cell extracts from *S. autotrophica* and *S. paralvinellae* and in cell suspensions of *S. denitrificans* (Timmer-Ten Hoor, 1981; Takai et al., 2005). Although *S. gotlandica* was described to be able to operate a dissimilatory sulfide oxidation pathway with APSR (cf. Grote et al., 2012), sequence analyses show that its APSR (Accession No. WP_008339777) contains only one subunit as it is typically found for the assimilatory sulfate reduction pathway (cf. Supplementary Figure S1D, see also above). The absence of the AprBA-type dissimilatory APSR indicates that *S. denitrificans* does not oxidize sulfite through this indirect pathway and thus ATPS likely only plays a role in assimilatory sulfate reduction (Sievert et al., 2008b). Moreso, despite the Sat-type ATPS being typically associated with dissimilatory sulfide oxidation (Neumann et al., 2000), we posit that this Sat-type ATPS is also involved in assimilatory sulfate reduction as also shown in Fungi and yeast (Foster et al., 1994; Ullrich et al., 2001; Frigaard and Dahl, 2008).

#### Polysulfide Reductase

Besides functioning as electron donors, sulfur compounds can also be electron acceptors. Although the solubility of elemental sulfur is quite low, polysulfide is formed by dissolving sulfur flower in an aqueous sulfide solution (Hedderich et al., 1998). One of *Sulfurimonas* species strain NS25-1, which was isolated from a deep-sea hydrothermal vent, can apparently grow with hydrogen as an electron donor and with elemental sulfur as an electron acceptor (Nakagawa et al., 2005b). However, there is no experimental evidence of sulfur reduction taking place in the cultivation. In another *Epsilonproteobacteria*, namely *Wolinella succinogenes*, polysulfide is a possible intermediate of sulfur reduction with hydrogen where electrons are transferred from a hydrogenase to the polysulfide reductase (PSR; Hedderich et al., 1998). A similar mechanism was also proposed in the epsilonproteobacterial *Nautilia profundicola*, isolated from a hydrothermal vent (Campbell et al., 2009).

All genome sequenced *Sulfurimonas* species harbor PSR-homologs on their genomes (Supplementary Table S1, Supplementary Figure S1G, Sievert et al., 2008b; Sikorski et al., 2010b; Grote et al., 2012; Cai et al., 2014) and we posit that the reduction of elemental sulfur coupled to hydrogen oxidation may also be prevalent in distinct *Sulfurimonas* species. Phylogenetic analysis shows that PSR catalytic subunits (PsrA) from non-vent *Sulfurimonas* species form a monophyletic group and are close to those from *Sulfuricurvum* species, which are terrestrial strains (Supplementary Figure S1G). The PsrA from the hydrothermal-vent isolate *S. autotrophica* is clustered with that of another vent isolate, i.e., *Nitratiruptor* sp SB155-2 (Supplementary Figure S1G). Hence, PSRs from the same environment but different epsilonproteobacterial genera appear to be more closely related than those from the same genera but different habitats.

### Hydrogen Metabolism

Most *Sulfurimonas* species are capable of growing with hydrogen as an energy source: *S. denitrificans, S. paralvinellae, S. gotlandica*, and *S. hongkongensis* (**Table 1**; Takai et al., 2006; Sievert et al., 2008b; Labrenz et al., 2013; Cai et al., 2014; Han and Perner, 2014). *S. denitrificans* and *S. hongkongensis* can grow with hydrogen and nitrate (Sievert et al., 2008b; Cai et al., 2014; Han and Perner, 2014). *S. paralvinellae* uses hydrogen as an electron donor in the presence of reduced sulfur compounds (Takai et al., 2006). Hydrogen utilization was also detected in *S. gotlandica* when it grew in medium with nitrate and thiosulfate (Labrenz et al., 2013). In contrast, *S. autotrophica* and *Thiomicrospira* sp. CVO have not been shown to grow with hydrogen under aerobic (6%) and anaerobic conditions, respectively (**Table 1**; Gevertz et al., 2000; Inagaki et al., 2003; Takai et al., 2006).

**FIGURE 3:**
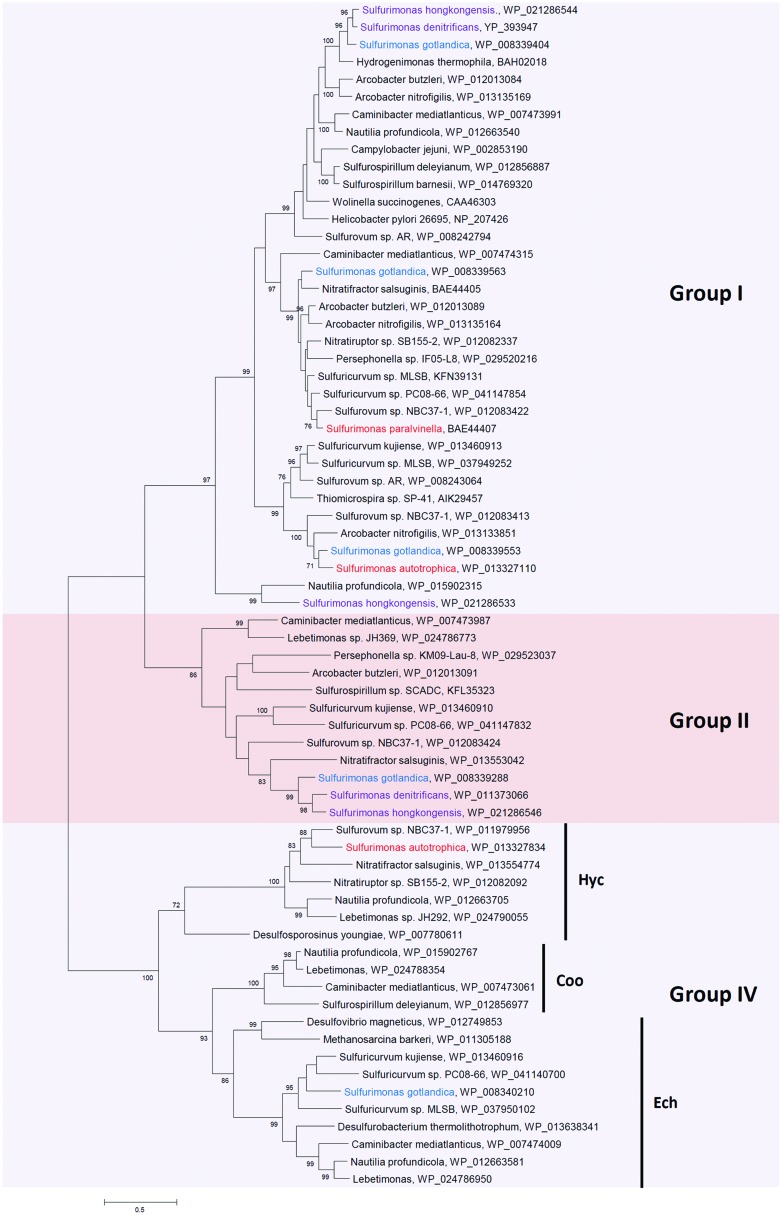
**Phylogenetic relationships of hydrogenase large subunit sequences to those from *Sulfurimonas* species**. The phylogenetic tree was constructed with the same method as described in **Figure 2**. The percentage of bootstrap resamplings ≥70 is indicated on the branches. The scale bar represents the expected number of changes per amino acids position. The groups of hydrogenases are classified according to Vignais and Billoud (2007). Isolation sources of *Sulfurimonas* species are indicated in different colors: blue, marine non-vent water system; purple, marine sediments; red, hydrothermal environments.

*Epsilonproteobacteria* mostly use the [NiFe]-hydrogenase to catalyze the reaction H_2_ ↔ 2H^+^ + 2e^-^ (Vignais and Billoud, 2007). The [NiFe]-hydrogenase is a heterodimer with a small subunit containing three iron–sulfur clusters and a large subunit containing the active site. They are classified in four groups, namely Groups I to IV (Vignais and Billoud, 2007). Except for some of the human and animal pathogens and *Thiovolum*, most *Epsilonproteobacteria* have a broad array of different [NiFe]-hydrogenases affiliated with Group I, II, and IV (Supplementary Table S1; **Figure 3**). Group I hydrogenases are membrane-bound hydrogenases performing respiratory hydrogen oxidation linked to the reduction of electron acceptors (Vignais and Billoud, 2007). They are found in all five *Sulfurimonas* species (**Figure 3**, Supplementary Table S1 and references therein), indicating that this group of hydrogenases might be essential for growth. The periplasmic Group I [NiFe]-hydrogenase of *S. denitrificans* contributes to hydrogen uptake activity in the membrane fractions of the cell extracts (Han and Perner, 2014). In *S. paralvinella* hydrogen uptake activity was also detected (Takai et al., 2005). However, for *S. gotlandica* and *S. hongkongensis* these experiments have not been performed to date. In line with lacking growth on hydrogen, no hydrogen uptake activity was detected in the cell extracts of *S. autotrophica* (Takai et al., 2005). However, since its genome harbors hydrogenases (Sikorski et al., 2010b), under specific environmental conditions *S. autotrophica* may well be able to express hydrogenases and consume hydrogen. *S. hongkongensis* and *S. gotlandica* even have two and three Group I [NiFe]-hydrogenases, respectively, which may reflect the enzymes’ abilities to function at different hydrogen concentrations, as has been suggested in the *Epsilonproteobacteria N. profundicola* and *S.* sp. NBC 37-1 (Nakagawa et al., 2007; Campbell et al., 2009). The Group I *Sulfurimonas* hydrogenases group into different clusters with phylogenetically diverse *Epsilonproteobacteria* (**Figure 3**). This may suggest multiple acquisitions or possibly repeated loss of these hydrogenases among the *Sulfurimonas* species.

Group II hydrogenases were not found in the hydrothermal deep-sea vent *Sulfurimonas* species, but were present in the isolates from marine water and sediments, i.e., *S. denitrificans, S. hongkongensis* and *S. gotlandica* (**Figure 3**). In these habitats hydrogen is less abundant than in hydrothermal vents, suggesting that this group of hydrogenases may be important under low hydrogen concentrations. Intriguingly, no hydrogen uptake activity in the soluble protein fractions of *S. denitrificans* was detected, even though the transcripts of the large subunit of the cytoplasmic Group II [NiFe]-hydrogenase was recovered by reverse transcription PCR (Han and Perner, 2014). Its role thus remains speculative: in other *Epsilonproteobacteria* it has been proposed to function as a hydrogen-sensing hydrogenase (Nakagawa et al., 2007). In contrast, Sievert et al. (2008b) posited that the cytoplasmic hydrogenase of *S. denitrificans* most likely functions as a catalytically active hydrogenase because of the sequence similarity of both subunits to the enzyme from *A. aeolicus*, where it is involved in the reverse electron transport whereby increasing efficiency of growth (Brugna-Guiral et al., 2003). Alternatively, it has been suggested that the Group II hydrogenase is important during phases of hydrogen and polysulfide starvation, where internal carbon storages are used up due to running the oxidative tricarboxylic acid cycle (Campbell et al., 2009). Reduced ferredoxin could be reoxidized by the hydrogenase whereby the cycle could continue and hydrogen could be produced favorably (Campbell et al., 2009). Further experiments will be necessary to confirm the actual role of the cytoplasmic hydrogenase for *S. denitrificans* and the other *Sulfurimonas* species that encode one.

*Sulfurimonas* differ from several other hydrogen metabolizing *Epsilonproteobacteria* in that they have very few Group IV hydrogenases (Supplementary Table S1), which are hydrogen-evolving, energy-conserving, membrane-associated multimeric enzymes (Vignais and Billoud, 2007). *S. autotrophica* and *S. gotlandica* have one Group IV [NiFe]-hydrogenase each, Hyc and Ech, respectively (**Figure 3**; Supplementary Table S1) (Sikorski et al., 2010b; Grote et al., 2012). Hyc from *S. autotrophica* is clustered with Hycs from other *Epsilonproteobacteria*, which are all isolated from hydrothermal deep-sea vents (**Figure 3**; Supplementary Table S1). Since hydrothermal vents are extremely acidic (pH 2-4) (Fisher et al., 2007), Hyc from vent isolates might be related to anaerobic acid resistance through proton conversion as proposed in anaerobic growing cultures of *Escherichia coli* (Noguchi et al., 2010). Here, Hyc catalyzes hydrogen generation with protons at low external pH (Noguchi et al., 2010). Another Group IV hydrogenase, Ech, has been well characterized in the archaeon *Methanosarcina barkeri*. It catalyzes hydrogen formation through reduced ferredoxin and plays a role in carbon fixation from CO_2_ and acetate (Meuer et al., 2002). Likewise, it may function in the same way in *S. gotlandica*.

### Nitrogen Metabolism

*S. denitrificans* was the first isolated *Sulfurimonas* species and was characterized as a denitrifier using thiosulfate as an electron donor and nitrate as an electron acceptor (Timmer-Ten Hoor, 1975). Numerous 16S rRNA gene sequences have been identified since from coastal marine sediments and the Baltic Sea that are closely related to *S. denitrificans* and appear to account for much of the autotrophic denitrification (Brettar et al., 2006; Shao et al., 2009; Zhang et al., 2009). This indicates that *S. denitrificans*-based denitrification plays an important role in nitrogen and carbon cycling in these and likely similar environments. Except for *S. autotrophica*, all strains including *Thiomicrospira* sp. CVO are known to grow with nitrate as an electron acceptor (Timmer-Ten Hoor, 1981; Voordouw et al., 1996; Inagaki et al., 2003; Takai et al., 2006; Grote et al., 2012; Cai et al., 2014). However, nitrate turnover in the distinct *Sulfurimonas* differs considerably: *S. denitrificans* uses nitrate up rapidly, namely 20 mM nitrate in three (with hydrogen) to six (without hydrogen) days during growth when coupled to thiosulfate-oxidation (Han and Perner, 2014). *S. gotlandica* uses roughly 1 mM nitrate within 9 days in the presence of thiosulfate (Mammitzsch et al., 2014) and *S. paralvinellae* utilizes ∼10 mM nitrate within 90 h in the presence of hydrogen and sulfur (Takai et al., 2006). *S. denitrificans, S. gotlandica*, and *Thiomicrospira* sp. CVO can additionally grow with nitrite as an electron acceptor (see **Table 1** for references). All genes required for the complete reduction of nitrate to nitrogen gas, i.e., nitrate reductases, nitrite reductases, nitric oxide reductases and nitrous oxide reductases, were found on the genomes of *S. denitrificans, S. hongkongensis, S. autotrophica*, and *S. gotlandica* (Sievert et al., 2008b; Sikorski et al., 2010b; Grote et al., 2012; Cai et al., 2014). However, even though the genome of *S. autotrophica* contains the *napAGHBFLD* operon (Sikorski et al., 2010b), *S. autotrophica* is incapable of growing at a concentration of 5 mM sodium nitrate under the tested conditions (Inagaki et al., 2003).

**FIGURE 4:**
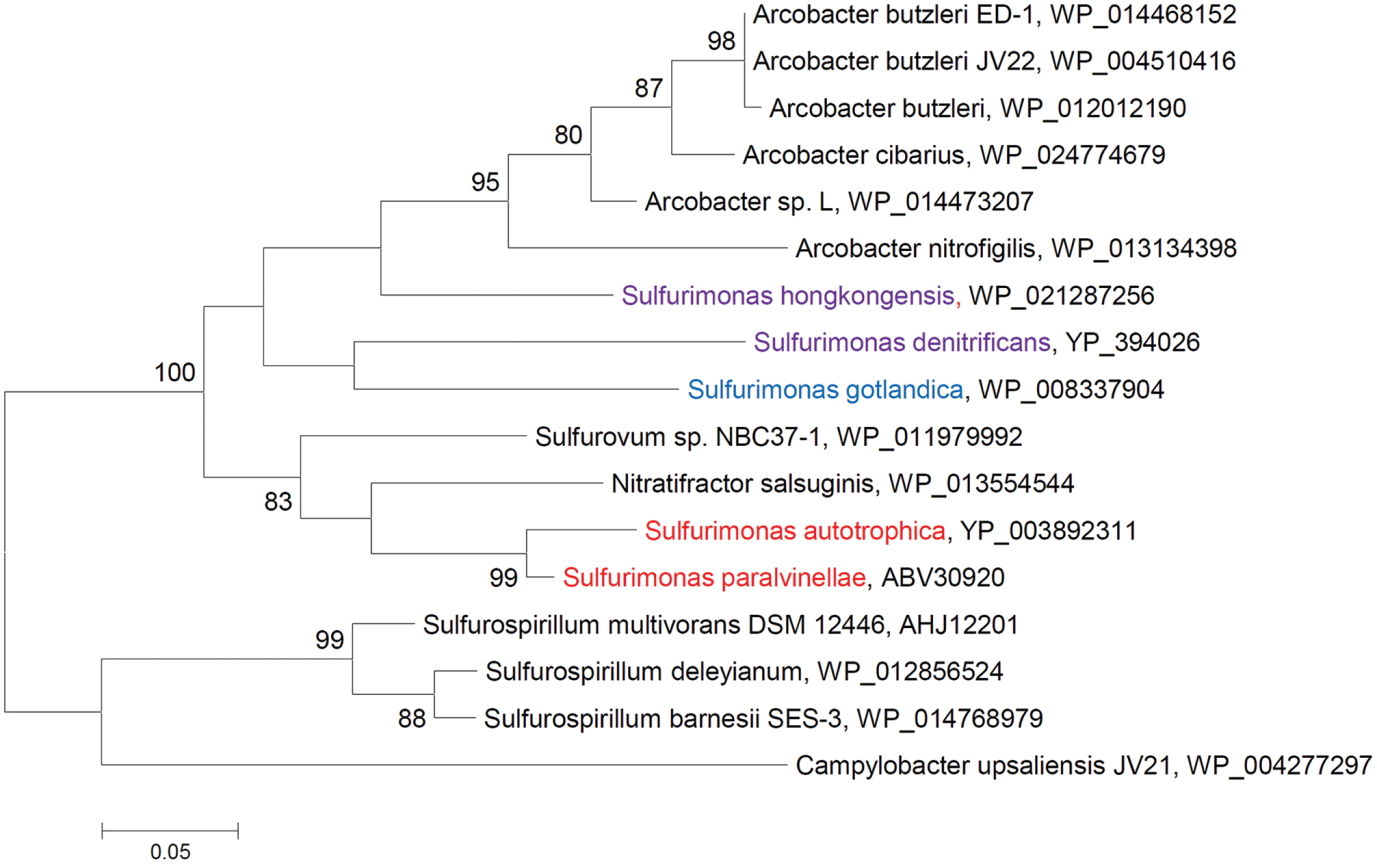
**Phylogenetic relationships of NapA sequences to those from *Sulfurimonas* species**. The phylogenetic tree was constructed with the same method as described in **Figure 2**. The percentage of bootstrap resamplings ≥70 is indicated on the branches. The scale bar represents the expected number of changes per amino acids position. Isolation sources of *Sulfurimonas* species are indicated in different colors: blue, marine non-vent water system; purple, marine sediments; red, hydrothermal environments.

All so far isolated and tested *Sulfurimonas* species have the periplasmic nitrate reductase (Nap) catalytic subunit (NapA) (**Figure 4**) and all so far genome sequenced *Sulfurimonas* species have the *napAGHBFLD* operon (Sievert et al., 2008b; Sikorski et al., 2010b; Grote et al., 2012; Cai et al., 2014). This organization of the *nap* operon is distinct from other proteobacterial *nap* operons (Potter et al., 2001), but is conserved in deep-sea hydrothermal vent *Epsilonproteobacteria* (Vetriani et al., 2014). Since such vent NapAs have a high affinity for nitrate, they may represent the adaption of *Sulfurimonas* species to the low nitrate concentrations in vent systems (Vetriani et al., 2014) and other nitrate low environments.

Interestingly, epsilonproteobacterial NapAs of isolates from hydrothermal vents clustered, while those from non-vent but marine habitats grouped and were closely related to *Arcobacter* species (**Figure 4**). Thus, the NapA proteins from the same habitats appear to be more similar to each other than to those from the same genus.

### Oxygen Reduction

Besides nitrate and nitrite, oxygen can also serve as an electron acceptor in most *Sulfurimonas* species and *Thiomicrospira* sp. CVO (**Table 1**). All four sequenced genomes of *Sulfurimonas* species contain a *ccoNOQP* operon (also called *fixNOQP*) encoding a *cbb*_3_-type cytochrome *c* oxidase (*cbb*_3_-COX) (**Table 2**, Sievert et al., 2008b; Sikorski et al., 2010b; Grote et al., 2012; Cai et al., 2014), which is a proton-pumping respiratory heme-copper oxidase involved in transferring electrons from cytochrome *c* to oxygen and reduction of oxygen to water (Pitcher and Watmough, 2004). Because of its high affinity for oxygen, it plays an important role in supporting bacterial growth under microaerobic conditions (Marchal et al., 1998; Bertini et al., 2005). Phylogenetic analysis of the *cbb*_3_-COX catalytic subunit (CcoN/FixN) shows that these subunits from *Sulfurimonas* species form a monophyletic cluster (Clade I in Supplementary Figure S1H), indicating that this type of *cbb*_3_-COXs is important for oxygen reduction in *Sulfurimonas* species. These type *Sulfurimonas*’ *cbb*_3_-COXs are clustered within CcoNs from non-*Sulfurimonas Epsilonproteobacteria*, which can also use oxygen as electron acceptors (Clade I in Supplementary Figure S1H), indicating that these *cbb*_3_-COXs are conserved enzymes throughout the group. Among *Epsilonproteobacteria* only *S. denitrificans, S. autotrophica*, and *S.* sp. NBC 37-1 contain an additional CcoN, which is located on different parts of the genome than the *ccoNOQP* operon (Nakagawa et al., 2007; Sievert et al., 2008b; Sikorski et al., 2010b). These second *Sulfurimonas*’ CcoNs are clustered with those from *Aquificales* and *Actinobacteria* (Clade II in Supplementary Figure S1H). The oxygen reductase activity of this type CcoN was demonstrated in *Aquificales*: *Sulfurihydrogenibium azorense* (Ducluzeau et al., 2008). The growth experiments illustrated that *S. denitrificans* and *S. autotrophica* can indeed use oxygen as an electron acceptor (Timmer-Ten Hoor, 1981; Inagaki et al., 2003), coinciding with the presence of a *ccoNOQP* operon on their genomes. In contrast, no growth of *S. gotlandica* with oxygen as a sole electron acceptor was detected, despite the *ccoNOQP* operon’s presence on its genome (Grote et al., 2012; Labrenz et al., 2013). Nevertheless, the authors claimed that *S. gotlandica* might be able to use oxygen as an electron acceptor but that the growth rate is too low for detection with their experimental design. In the presence of oxygen, *S. gotlandica* can still grow using nitrate as an electron acceptor. When the oxygen concentration is below 10%, *S. gotlandica*’s growth is not affected in the medium (with nitrate and thiosulfate), but growth is considerably impaired when the oxygen concentration exceeds 20% (Labrenz et al., 2013). This phenomenon can be explained by the induction of *ccoN* expression with low oxygen tension (Preisig et al., 1993; Mouncey and Kaplan, 1998) and its possible function as an oxygen scavenger to protect oxygen sensitive enzymes (Sievert et al., 2008b). Under low oxygen conditions, the expression of *ccoN* is induced and the activity of *cbb*_3_-COX is higher than under high oxygen conditions, diminishing the amount of oxygen and thereby protecting the oxygen-labile enzymes. Growth of *S. gotlandica* with nitrate and thiosulfate can then proceed. In contrast, under high oxygen concentrations the activity of *cbb*_3_-COX is lower, the activities of oxygen-sensitive enzymes for the growth with nitrate and thiosulfate are reduced, resulting in less growth. *S. hongkongensis* was defined as a strict anaerobe (Cai et al., 2014) and there is no more information about its usage of oxygen. However, apparently *S. hongkongensis* survived after being exposed to air at 4°C for up to 8 weeks (personal communication with M. Shao and T. Zhang), which does suggest some oxygen tolerance. Nevertheless, since it has the same type of *ccoNOQP* operon and it lacks the same additional *ccoN* gene as *S. gotlandica*, we expect it to behave in a similar way to oxygen as *S. gotlandica* does.

**Table 2:** List of key enzymes for sulfur, nitrogen, hydrogen, and carbon metabolisms in *Sulfurimonas* species.

	*S. denitrificans*	*S. hongkongensis*	*S. autotrophica*	*S. paralvinellae*	*S. gotlandica*	Phylogenic tree of catalytic subunits
Genome size	2.20 Mbp	2.30 Mbp	2.15 Mbp	NR	2.95 Mbp	
Sulfur metabolisms						
Sulfide:quinone reductase (SQR)	**+** (3)	**+** (5)	**+** (4)	NR	**+** (5)	**Figure 2**
Sulfur oxidation protein (Sox)	**+**	**+**	**+**	NR	**+**	Supplementary Figure S1A
Sulfite dehydrogenase (SOR)	**-**	**+**	**+**	NR	**+**	Supplementary Figure S1B
adenylylsulfate reductase (APSR)	**-**	**-**	**-**	NR	**-**	
ATP sulfurylase (ATPS, ATP:sulfate adenylyltransferase)	**+** (2)	**+**	**+** (2)	NR	**+** (3)	Supplementary Figure S1C
Nitrogen metabolisms						
Periplasmic nitrate reductase (Nap)	**+**	**+**	**+**	**+**	**+**	**Figure 4**
Cytoplasmic nitrate reductase (Nas)	**-**	**-**	**+**	NR	**+**	
Hydrogen metabolism						
Hydrogenase	**+** (2)	**+** (3)	**+** (2)	**+**	**+** (5)	**Figure 3**
Carbon metabolisms						
Oxoglutarate:ferredoxin oxidoreductase (OOR)	**+**	**+**	**+**	**+**	**+**	
Pyruvate:ferredoxin oxidoreductase (POR)	**+**	**+**	**+**	NR	**+**	
ATP-dependent citrate lyase (ACL)	**+**	**+**	**+**	**+**	**+**	Supplementary Figure S1I
Oxygen reduction						
*cbb*_3_-type cytochrome *c* oxidase (COX)	**+** (2)	**+**	**+** (2)	NR	**+**	Supplementary Figure S1H
Reference	Sievert et al., 2008b	Cai et al., 2014	Sikorski et al., 2010b	Takai et al., 2005; Vetriani et al., 2014	Grote et al., 2012	

+, present; the number of enzymes are listed in the brackets; -, not present; NR, not reported. ^*^, Thiomicrospira sp. CVO is not listed in this table, because its genome sequence is not available and none of the genes have been PCR-amplified.

### Autotrophic Carbon Dioxide Fixation

Growth experiments revealed that *Sulfurimonas* species fix carbon dioxide autotrophically via the reductive tricarboxylic acid (rTCA) cycle. This is supported through genetic and enzymatic studies (Hügler et al., 2005; Takai et al., 2005; Sievert et al., 2008b; Grote et al., 2012; Cai et al., 2014). Genes encoding key enzymes, e.g., 2-oxoglutarate:ferredoxin oxidoreductase (OOR), pyruvate:ferredoxin oxidoreductase (POR), and ATP-dependent citrate lyase (ACL), for the reductive citric acid cycle are found on the genomes of *S. denitrificans, S. autotrophica, S. gotlandica*, and *S. paralvinellae* either by genome sequencing or PCR amplification (Supplementary Figure S1I; **Table 2**, Takai et al., 2005; Sievert et al., 2008b; Sikorski et al., 2010b; Grote et al., 2012). The activities of OORs, PORs, and ACLs were detected in the cell extracts of *S. denitrificans* (Hügler et al., 2005), *S. autotrophica* and *S. paralvinellae* (Takai et al., 2005). It is known that the rTCA cycle genes are very abundant in the sequences from deep-sea hydrothermal vents (Campbell and Cary, 2004). Phylogenetic analysis shows that AclBs from *Sulfurimonas* species form a monophyletic group (Supplementary Figure S1I), suggesting this pathway might be conserved in *Sulfurimonas* species. AclBs from hydrothermal vent isolates *S. autotrophica* and *S. paralvinellae* are clustered together and AclBs from non-vent-isolated *Sulfurimonas* species are clustered together, indicating that different environments influence the evolution of AclB proteins.

### Usage of Organic Compounds

Although *Sulfurimonas* species are characterized as chemolithoautotrophs, some organic compounds were found to contribute to their growth (**Table 1**). For example, *S. paralvinellae* can use yeast extract besides reduced sulfur species as sulfur source (Takai et al., 2006). *Thiomicrospira* sp. CVO and *S. gotlandica* can use acetate in addition to carbon dioxide and bicarbonate as a carbon source (**Table 1**, Gevertz et al., 2000; Kodama and Watanabe, 2003; Labrenz et al., 2013). Aside from inorganic compounds, *S. denitrificans* can grow with formate, fumarate, yeast extract, alcohol mix as electron donors and *S. gotlandica* can grow with formate, acetate, yeast extract, pyruvate, and amino acid mix as electron donors (Labrenz et al., 2013). The ability of *Sulfurimonas* species to additionally exploit organic compounds suggests that those versatile metabolic strategies max contribute to the successful colonization of a broad type of environments.

## Structural Genes of Key Energy Metabolisms and Horizontal Gene Transfer

Of the investigated structural proteins from *Sulfurimonas* species the SoxBs, CcoNs (Clade I) and AclBs form monophyletic groups within the *Epsilonproteobacteria* (**Figure 2**; Supplementary Figures S1A,H,I) suggesting that these genes are vital for the cells’ survival and likely originate from a common ancestor. However, other genes of key pathways are phylogenetically positioned with non-*Sulfurimonas Epsilonproteobacteria* or non-*Epsilonproteobacteria*: e.g., Type III and Type V SQRs are clustered with SQRs from mesophilic bacteria (including a group of pathogens), thermophilic bacteria (e.g., *Sulfobacillus*) and thermophilic or/and acidophilic archaea, e.g., *Archaeoglobus, Pyrobaculum*, and some *Sulfolobales* and *Thermoplasmatales* (**Figure 2**); and CcoNs (Clade II) from two *Sulfurimonas* species cluster with CcoNs from the hydrothermal vent isolates *S.* sp. NB C37-1, *Aquificales* and *Actinobacteria* (Supplementary Figure S1H). The distribution of the structural protein NapAs and PsrAs from *Sulfurimonas* species is somehow different: NapAs from the hydrothermal vent isolated *Sulfurimonas* species and the ones from non-vent isolates form a clade each (**Figure 4**). NapA proteins from the two hydrothermal vent isolates *S. paralvinellae* and *S. autotrophica* are clustered with other deep-sea vent *Epsilonproteobacteria* (e.g., *Sulfurovum* and *Nitratifractor*) and NapA proteins from *Sulfurimonas* species isolated from marine non-vent sediments and the water column are clustered with pathogenic *Epsilonproteobacteria Arcobacter* species (**Figure 4**). Hence, NapA proteins from the hydrothermal vent organisms *Sulfurovum, Nitratifractor*, and *Sulfurimonas* are more similar to each other than NapAs from other marine *Sulfurimonas* species. The same pattern is also observed for PsrA proteins (Supplementary Figure S1G). In summary, these phylogenies strongly indicate the large role that horizontal gene acquisitions (from archaea, *Aquificales, Actinobacteria, Gammaproteobacteria*, and other *Epsilonproteobacteria*) appear to have had for shaping *Sulfurimonas*’ genomes and physiological properties in successfully surviving and flourishing in chemically distinct environments. This conclusion is consistent with the finding that besides conserved core genes, *Epsilonproteobacteria* generally also contain niche-specific genes, which are important to their metabolic diversity and versatility (Zhang and Sievert, 2014).

## Global Distributions and Metabolisms

*Epsilonproteobacteria* can colonize a broad range of habitats ranging from animal and human bodies, to terrestrial and marine environments, which include shallow waters and sediments but also deep sea hydrothermal vents (cf. Supplementary Table S1 and references therein). However, in the chemically distinct types of habitats that are inhabited by members of this lineage, the genomic inventory can differ considerably: particularly among the genes encoding SoxB (thiosulfate oxidation), SorA (sulfite oxidation), SQR (sulfide oxidation) and hydrogenases (both hydrogen uptake and evolution) (see Supplementary Table S1). Noticeably, the genomes of strains like the heterotrophic *Helicobacter, Campylobacter*, and *Wolinella*, which are limited to one type of habitat, namely animals and humans, appear relatively depleted in certain energy metabolisms. Nakagawa et al. (2007) have recently suggested that members of these groups lost hydrogen sensing and evolving hydrogenases as well as the ability to fix CO_2_ autotrophically because they have adapted themselves to their lifestyle, in case of *Helicobacter* and *Campylobacter* a pathogenic lifestyle, and do not depend on a diverse suit of metabolisms any longer.

Besides these heterotrophs colonizing only a comparable narrow habitat range, other epsilonproteobacterial heterotrophs exist, which have a broader global distribution: heterotrophic *Arcobacter* and *Sulfurospirillum* have been isolated from globally widespread and chemically diverging biotopes including terrestrial and marine provinces (Supplementary Table S1). While the *Arcobacter* and *Sulfurospirillum* listed in Supplementary Table S1 were isolated from humans, marshland, freshwater marsh and mud, forest ponds (McClung et al., 1983; Stolz et al., 1999; Miller et al., 2007; Sikorski et al., 2010a), their genomes still appear relatively limited with respect to key enzymes of different sulfur metabolizing pathways. This is likely related to the environments they were isolated from, either rich in organic compounds or rich in elemental sulfur. For example, some *Arcobacter* species were isolated from organic rich crude-oil contaminated sea water, petroleum reservoir, animals and plants (Kiehlbauch et al., 1991; Snaidr et al., 1997; Frias-Lopez et al., 2002; Grabowski et al., 2005; Prabagaran et al., 2007). Therefore, they are adapted to using different organic compounds from their habitats as energy sources (e.g., dimethyl sulfoxide as electron acceptor) and carbon sources (e.g., organic and amino acids) (Miller et al., 2007). It will be interesting to sequence the genome of the only so far isolated autotrophic *Arcobacter* (Wirsen et al., 2002) and compare the genetic inventory with that from the heterotrophic *Arcobacter*. *Sulfurospirillum deleyianum* was isolated from an anaerobic enrichment culture targeting *Desulfuromonas* where a medium that was rich in elemental sulfur was used (Wolfe and Penning, 1977). Thus, in the latter case, sulfur reduction appears to be more important for growth than oxidation of reduced sulfur compounds. In contrast, these genera can harbor a relatively broad suit of hydrogenases (Supplementary Table S1), which may catalyze hydrogen oxidation coupled to the reduction of various electron acceptors, including nitrate, oxygen, elemental sulfur, and organic compounds, e.g., fumarate (McClung et al., 1983; Stolz et al., 1999; Miller et al., 2007). Likewise, the anaerobic hydrogen oxidizers, which have so far only been isolated from hydrothermal vents, namely *Caminibacter, Nautilia*, and *Lebetimonas*, exhibit an exceptional high number of distinct hydrogenases (Supplementary Table S1). However, they appear to be relatively limited in their inventory for key enzymes of sulfur oxidizing metabolisms, which coincides with their physiology (Voordeckers et al., 2005; Smith et al., 2008; Campbell et al., 2009; Giovannelli et al., 2011; Meyer and Huber, 2014).

The broadest suit of genes encoding for enzymes capable of oxidizing thiosulfate, sulfite and sulfide as well as hydrogen uptake and evolution can be found among autotrophic, aerobic sulfur oxidizing species, primarily among *Sulfurimonas, Sulfurovum*, and *Sulfuricurvum* (Nakagawa et al., 2007; Sievert et al., 2008b; Sikorski et al., 2010b; Grote et al., 2012; Han et al., 2012; Park et al., 2012; Cai et al., 2014; Hamilton et al., 2014; Tan and Foght, 2014). *Sulfurimonas, Sulfuricurvum*, and *Sulfurovum* have been isolated from marine sediments and hydrothermal vents, whereas *Sulfurimonas* and *Sulfuricurvum* have additionally been found in terrestrial systems (NCBI database, Timmer-Ten Hoor, 1975; Voordouw et al., 1996; Inagaki et al., 2003; Kodama and Watanabe, 2004; Takai et al., 2006; Nakagawa et al., 2007; Park et al., 2012; Labrenz et al., 2013; Cai et al., 2014; Hamilton et al., 2014; Tan and Foght, 2014). In these type of habitats, sulfide concentrations range from being undetectable up to several milimolar (Groenendaal, 1979; Brettar and Rheinheimer, 1992; Gevertz et al., 2000; Nakagawa et al., 2005a). All these three genera have Type IV and VI SQRs (Supplementary Table S1), likely to cope with different concentrations of sulfide, such as free sulfide in hydrothermal vents and metal sulfide in marine sediments or contaminated landmasses. Additionally, they have one or more copies of Type II SQRs (Supplementary Table S1), which may compensate for the function of other SQRs under environmental conditions, where folding of SQRs in the periplasm is becoming increasingly difficult and thus no functional SQR enzyme would otherwise be available. In case of *Sulfurimonas*, some species harbor Type III and V SQRs besides Type II, IV, and VI SQRs (Supplementary Table S1), which may either enhance the energy generation with sulfide or have different functions, such as detoxification and sulfide signaling. With respect to hydrogen metabolism, these three broadly distributed genera harbor multiple copies of Group I hydrogenases for hydrogen uptake (Supplementary Table S1), likely exhibiting different hydrogen affinities, e.g., the Gotland Deep in the central Baltic Sea, where *S. gotlandica* was isolated from, is known to be a highly dynamic environment (Brettar and Rheinheimer, 1992; Grote et al., 2012; Labrenz et al., 2013). They also encode Group II hydrogenases, possibly involved in hydrogen sensing or energy conversion at low hydrogen concentrations (Sievert et al., 2008b; Campbell et al., 2009). Additionally they harbor Group IV hydrogenases for hydrogen evolution, probably coupling with proton conversion in hydrothermal vents or with energy conversion through carbon fixation. However, unfortunately, there is no experimental evidence to provide a precise understanding of the distinct SQR and hydrogenase functions and their roles remain enigmatic in these groups. Hence, it will be essential to investigate the roles of the different types of SQRs and hydrogenases in these (and other epsilonproteobacterial) genera to better understand how they deal with distinct environmental conditions. This knowledge would also shed light on how the environmental conditions can shape species’ physiology.

## Conflict of Interest Statement

The authors declare that the research was conducted in the absence of any commercial or financial relationships that could be construed as a potential conflict of interest.
