# Supplementary material for: The globally widespread genus Sulfurimonas: versatile energy metabolisms and adaptations to redox clines
Source: Front Microbiol. 2015 Sep 16;6:989. doi: 10.3389/fmicb.2015.00989 (PMC4584964; doi:10.3389/fmicb.2015.00989)
Supplement: Supplementary file 1 [file Data_Sheet_1.PDF]

Supplementary Material

The globally widespread genus *Sulfurimonas*: versatile energy metabolisms and adaptations to global redox cline

Yuchen Han and Mirjam Perner*

Molecular Biology of Microbial Consortia, University of Hamburg, Biocenter Klein Flottbek, Hamburg, Germany

*** Correspondence:** Mirjam Perner, Molecular Biology of Microbial Consortia, Biocenter Klein Flottbek, University of Hamburg, Ohnhorststr. 18, 22609 Hamburg, Germany. Tel: +49-40-42816-444, Fax: +49-40-42816-459, E-mail: mirjam.perner@uni-hamburg.de

1. Supplementary Table S1

Supplementary Table S1. List of enzymes for sulfur and hydrogen metabolisms in representative strains within *Epsilonproteobacteia*. The genome sequences were acquired from NCBI databases, and the sequences of PsrAs, SoxBs, SorAs, SQRs and hydrogenses were compared with the non-redundant protein database of NCBI by using BLASTP.

**Genus**	**Habitats**	**Representative strain**	**PsrA**	**SoxB**	**SorA**	**SQR**					**Hydrogenase**			**Reference**
						II	III	IV	V	VI	I	II	IV	
Nautilia	hydrothermal vent	*N. profundicola*	1	-	-	-	-	-	-	-	2	1	4	(Campbell et al., 2009)
Caminibacter	hydrothermal vent	*C. mediatlanticus*	1	-	-	-	-	1	-	1	2	1	4	(Giovannelli et al., 2011)
Lebetimonas	hydrothermal vent	*L.* sp. JH369	1	-	-	-	-	1	-	-	1	1	4	(Meyer and Huber, 2014)
Nitratiruptor	hydrothermal vent	*N.* sp. SB155-2	1	1	-	1	-	1	-	1	1	1	1	(Nakagawa et al., 2007)
Nitratifractor	hydrothermal vent	*N. salsuginis*	1	1	-	1	-	1	-	1	1	1	1	(Anderson et al., 2011)
Sulfurovum	hydrothermal vent and marine sediment	*S.* sp. NBC37-1	1	1	2	4	-	1	-	1	2	1	2	(Nakagawa et al., 2007)

		S. sp. AR	1	1	1	1	-	1	-	1	2	-	-	(Park et al., 2012)
Sulfuricurvum	terrestrial oil field, rice mill, coal-tar source, aquifer and hydrothermal vent	*S. kujiense*	1	1	2	2	-	1	-	1	2	1	1	(Han et al., 2012)
		S. sp. MLSB	1	1	1	1	-	1	-	1	2	1	1	(Tan and Foght, 2014)
		S. sp. PC08-66	1	1	-	1	-	1	-	1	1	1	1	(Hamilton et al., 2014)
Arcobacter	animal and human pathogen, hydrothermal vent, estuarine sediment, marine water, food, sewage and root of the marsh plant	*A. butzleri* RM4018	-	1	-	1	-	-	-	-	2	1	1	(Miller et al., 2007)
		A.nitrofigilis	1	1	-	-	-	1	-	1	3	1	1	(Pati et al., 2010)
Helicobacter	animal and human pathogen	*H. pylori* 26695	-	-	-	-	-	-	-	-	1	-	-	(Tomb et al., 1997)
Campylobacter	animal and human pathogen	*C. jejuni* ATCC700819	-	-	-	-	-	-	-	-	1	-	-	(Parkhill et al., 2000)
Sulfurospirillum	marine sediments, hydrothermal vent and terrestrial environments	*S. barnesii*	1	-	-	1	-	-	-	-	1	-	2	(Lucas et al., 2015)
		S. deleyianum	1	-	-	1	-	-	-	-	1	-	2	(Sikorski et al., 2010a)
Thiovulum	slough, meromictic lake and marine sediment	*T.* sp. ES	1	-	-	-	-	1	-	1	-	-	1	(Marshall et al., 2012)
Wolinella	animal and human	*W. succinogenes*	1	-	-	1	-	-	-	-	1	-	1	(Baar et al., 2003)
Sulfurimoans	coastal marine sediment, marine water, hydrothermal vent and terrestrial oil field	*S. autotrophica*	1	1	1	1	-	1	1	1	1	-	1	(Sikorski et al., 2010b)
		S. paralvinellae	NR	NR	NR	NR	NR	NR	NR	NR	1	NR	NR	(Takai et al., 2005)
		S. denitrificans	1	1	-	1	1	1	-	-	1	1	-	(Sievert et al., 2008)
		S. hongkongensis	1	1	1	2	1	1	-	1	2	1	-	(Cai et al., 2014)
		S. gotlandica	1	1	1	2	1	1	-	1	3	1	1	(Grote et al., 2012)

Numbers, the number of enzymes; -, not present; NR, not reported.

2. Supplementary Figure S1

A. SoxB

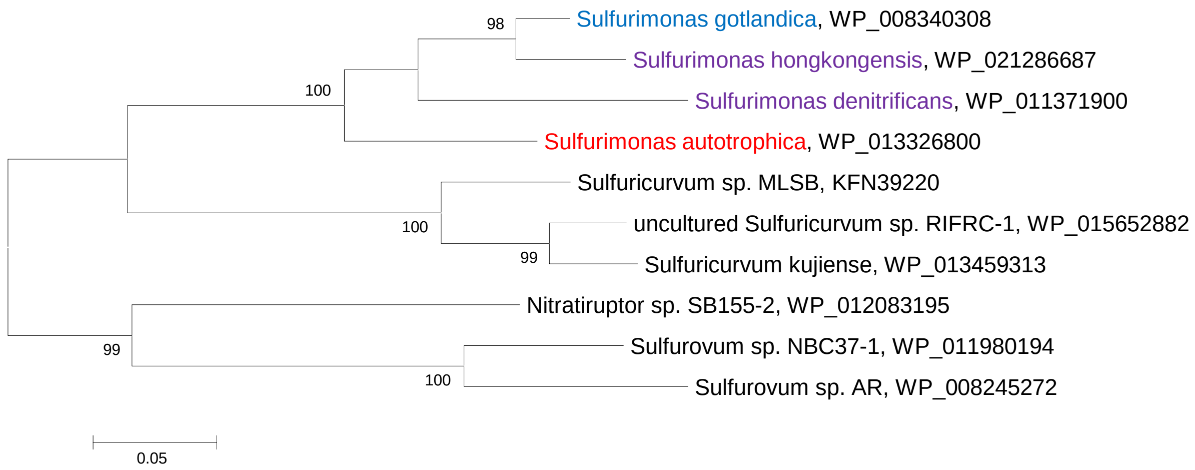

B. SorA

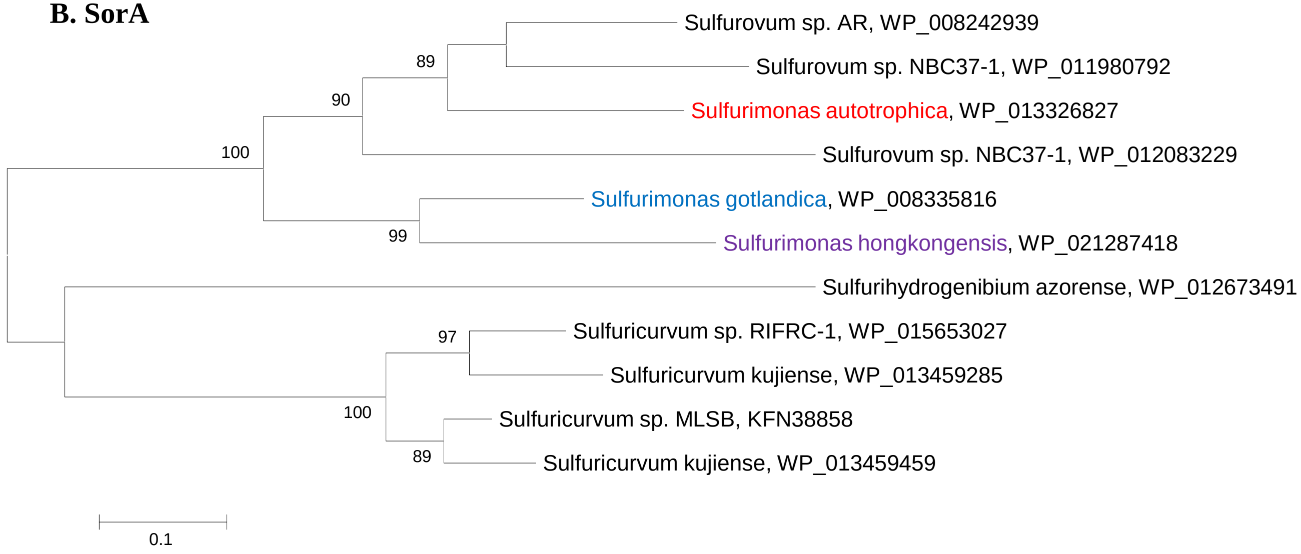

C. ATPS

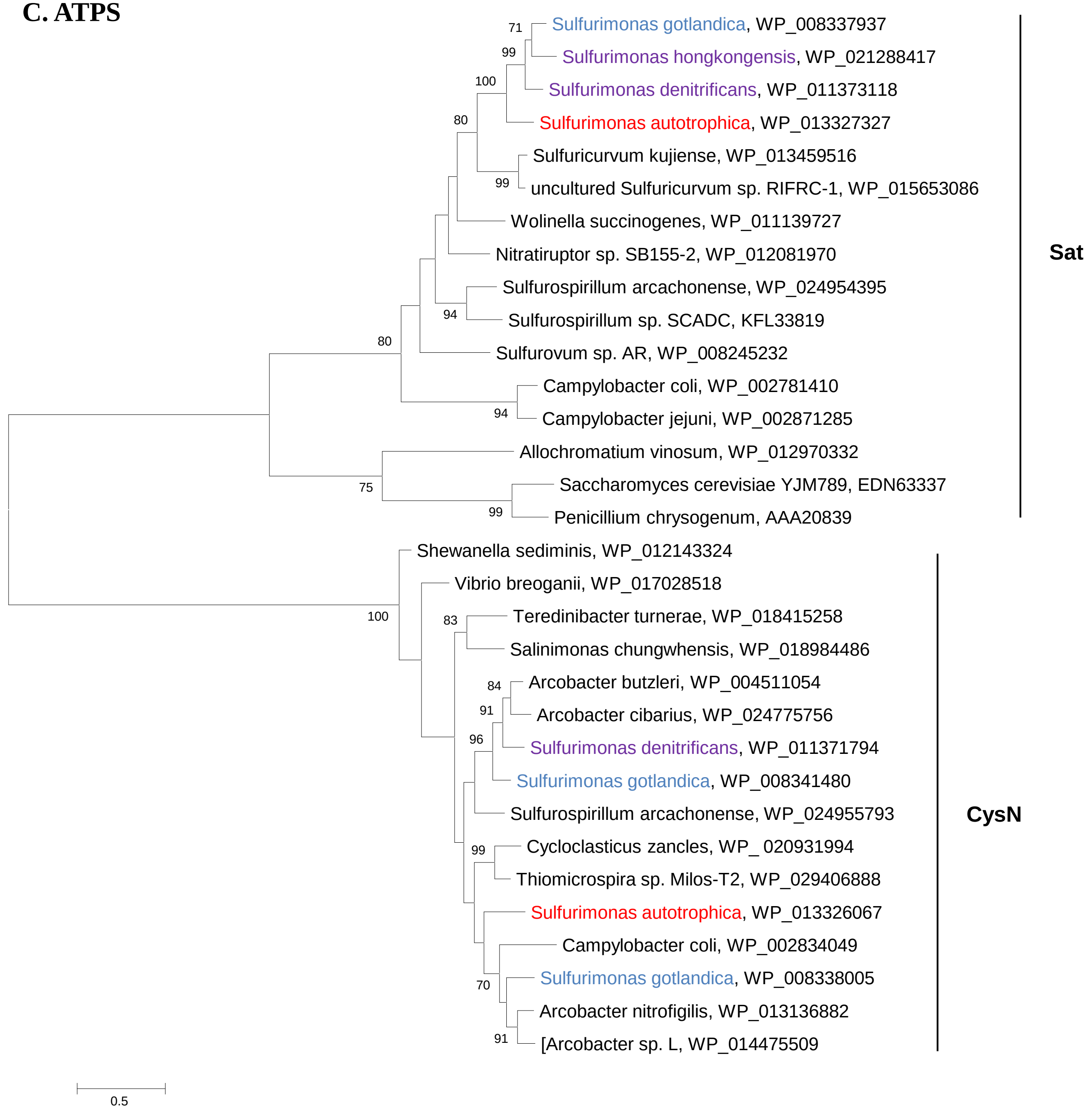
Sulfurimonas gotlandica, WP_008337937
Sulfurimonas hongkongensis, WP_021288417
Sulfurimonas denitrificans, WP_011373118
Sulfurimonas autotrophica, WP_013327327
Sulfuricurvum kujiense, WP_013459516
uncultured Sulfuricurvum sp. RIFRC-1, WP_015653086
Wolinella succinogenes, WP_011139727
Nitratiruptor sp. SB155-2, WP_012081970
Sulfurospirillum arcachonense, WP_024954395
Sulfurospirillum sp. SCADC, KFL33819
Sulfurovum sp. AR, WP_008245232
Campylobacter coli, WP_002781410
Campylobacter jejuni, WP_002871285
Allochromatium vinosum, WP_012970332
Saccharomyces cerevisiae YJM789, EDN63337
Penicillium chrysogenum, AAA20839
Sat
Shewanella sediminis, WP_012143324
Vibrio breoganii, WP_017028518
Teredinibacter turnerae, WP_018415258
Salinimonas chungwhensis, WP_018984486
Arcobacter butzleri, WP_004511054
Arcobacter cibarius, WP_024775756
Sulfurimonas denitrificans, WP_011371794
Sulfurimonas gotlandica, WP_008341480
Sulfurospirillum arcachonense, WP_024955793
Cycloclasticus zancles, WP_ 020931994
Thiomicrospira sp. Milos-T2, WP_029406888
Sulfurimonas autotrophica, WP_013326067
Campylobacter coli, WP_002834049
Sulfurimonas gotlandica, WP_008338005
Arcobacter nitrofigilis, WP_013136882
[Arcobacter sp. L, WP_014475509
CysN
71
99
100
80
99
94
80
94
75
99
100
83
84
91
96
99
70
91
0.5

D. CysH

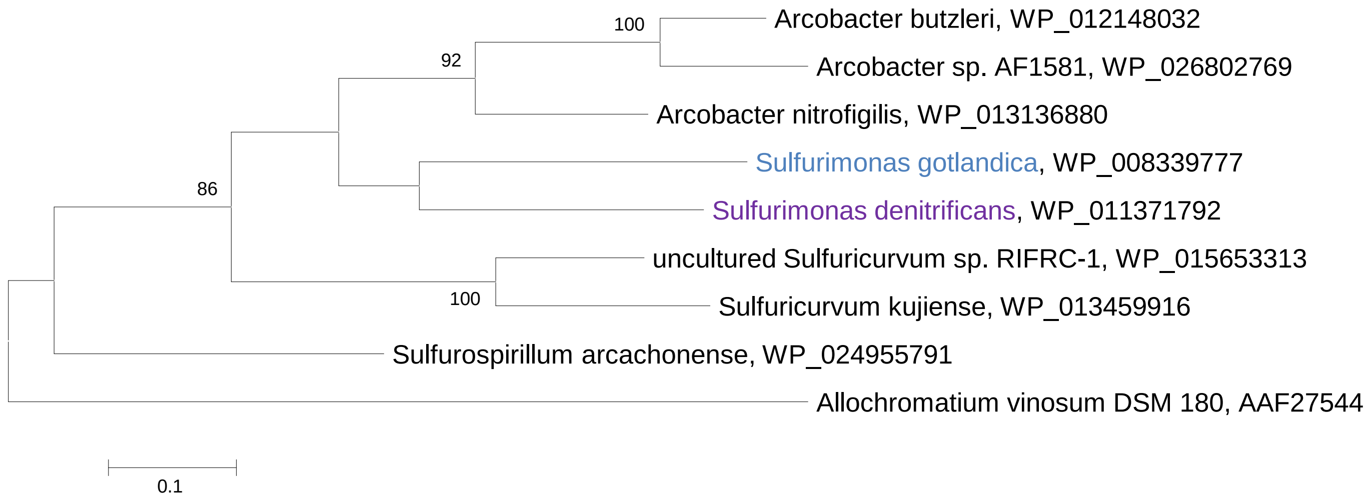
100
92
86
100
Arcobacter butzleri, WP_012148032
Arcobacter sp. AF1581, WP_026802769
Arcobacter nitrofigilis, WP_013136880
Sulfurimonas gotlandica, WP_008339777
Sulfurimonas denitrificans, WP_011371792
uncultured Sulfuricurvum sp. RIFRC-1, WP_015653313
Sulfuricurvum kujiense, WP_013459916
Sulfurospirillum arcachonense, WP_024955791
Allochromatium vinosum DSM 180, AAF27544
0.1

E. CysI

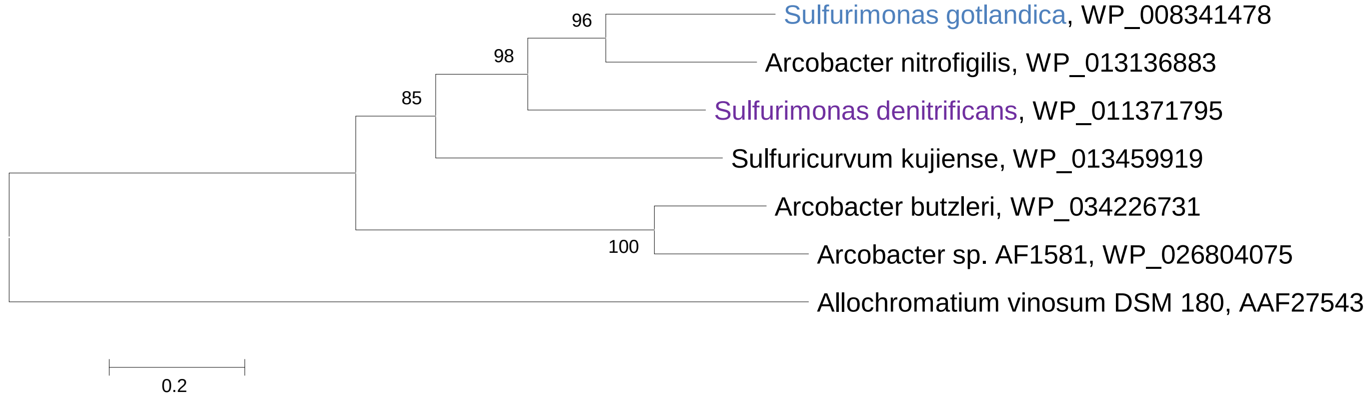
96
98
85
100
Sulfurimonas gotlandica, WP_008341478
Arcobacter nitrofigilis, WP_013136883
Sulfurimonas denitrificans, WP_011371795
Sulfuricurvum kujiense, WP_013459919
Arcobacter butzleri, WP_034226731
Arcobacter sp. AF1581, WP_026804075
Allochromatium vinosum DSM 180, AAF27543
0.2

F. CysC

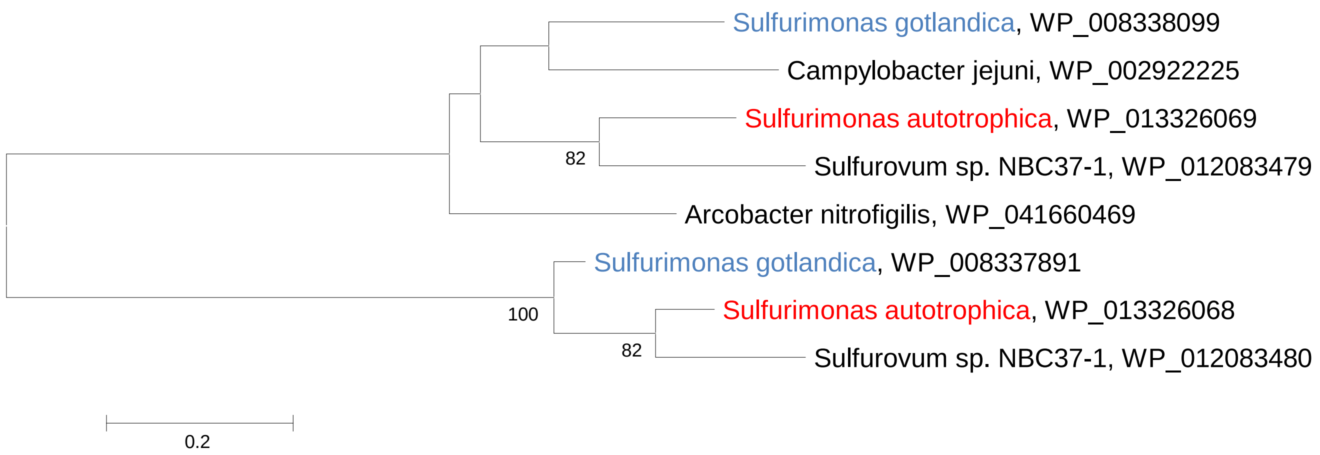
Sulfurimonas gotlandica, WP_008338099
Campylobacter jejuni, WP_002922225
Sulfurimonas autotrophica, WP_013326069
82
Sulfurovum sp. NBC37-1, WP_012083479
Arcobacter nitrofigilis, WP_041660469
Sulfurimonas gotlandica, WP_008337891
100
Sulfurimonas autotrophica, WP_013326068
82
Sulfurovum sp. NBC37-1, WP_012083480
0.2

G. PsrA

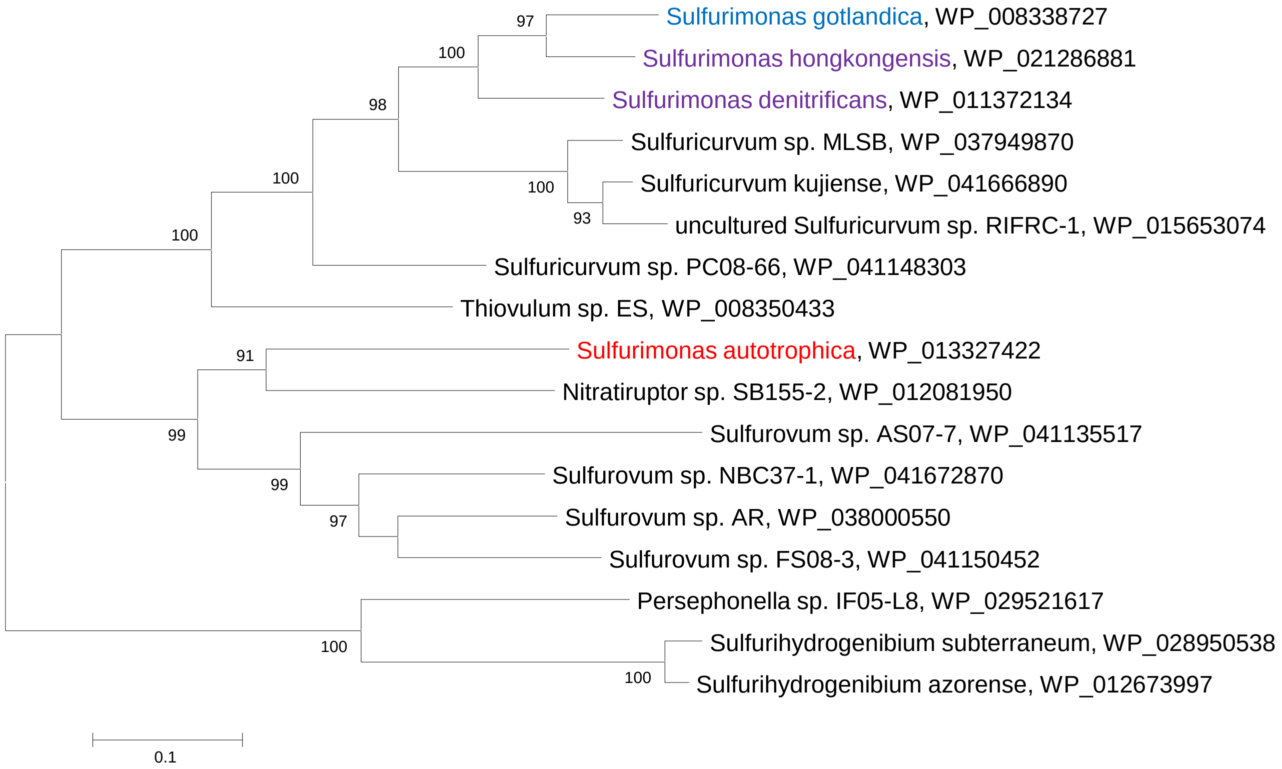
Sulfurimonas gotlandica, WP_008338727
Sulfurimonas hongkongensis, WP_021286881
Sulfurimonas denitrificans, WP_011372134
Sulfuricurvum sp. MLSB, WP_037949870
Sulfuricurvum kujiense, WP_041666890
uncultured Sulfuricurvum sp. RIFRC-1, WP_015653074
Sulfuricurvum sp. PC08-66, WP_041148303
Thiovulum sp. ES, WP_008350433
Sulfurimonas autotrophica, WP_013327422
Nitratiruptor sp. SB155-2, WP_012081950
Sulfurovum sp. AS07-7, WP_041135517
Sulfurovum sp. NBC37-1, WP_041672870
Sulfurovum sp. AR, WP_038000550
Sulfurovum sp. FS08-3, WP_041150452
Persephonella sp. IF05-L8, WP_029521617
Sulfurihydrogenibium subterraneum, WP_028950538
Sulfurihydrogenibium azorense, WP_012673997
97
100
98
100
100
93
100
91
99
99
97
100
100
0.1

H. CcoN

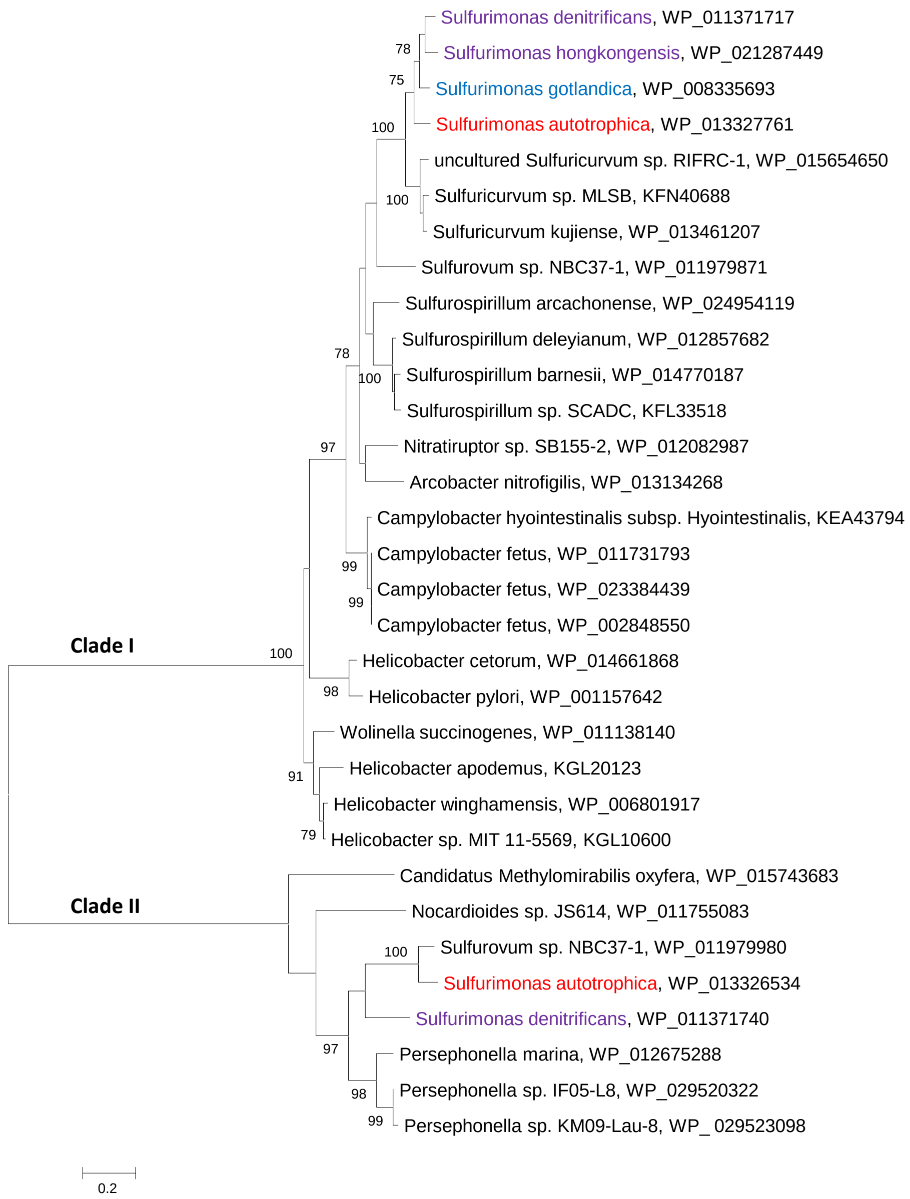
Sulfurimonas denitrificans, WP_011371717
Sulfurimonas hongkongensis, WP_021287449
Sulfurimonas gotlandica, WP_008335693
Sulfurimonas autotrophica, WP_013327761
uncultured Sulfuricurvum sp. RIFRC-1, WP_015654650
Sulfuricurvum sp. MLSB, KFN40688
Sulfuricurvum kujiense, WP_013461207
Sulfurovum sp. NBC37-1, WP_011979871
Sulfurospirillum arcachonense, WP_024954119
Sulfurospirillum deleyianum, WP_012857682
Sulfurospirillum barnesii, WP_014770187
Sulfurospirillum sp. SCADC, KFL33518
Nitratiruptor sp. SB155-2, WP_012082987
Arcobacter nitrofigilis, WP_013134268
Campylobacter hyointestinalis subsp. Hyointestinalis, KEA43794
Campylobacter fetus, WP_011731793
Campylobacter fetus, WP_023384439
Campylobacter fetus, WP_002848550
Helicobacter cetorum, WP_014661868
Helicobacter pylori, WP_001157642
Wolinella succinogenes, WP_011138140
Helicobacter apodemus, KGL20123
Helicobacter winghamensis, WP_006801917
Helicobacter sp. MIT 11-5569, KGL10600
Candidatus Methylomirabilis oxyfera, WP_015743683
Nocardioides sp. JS614, WP_011755083
Sulfurovum sp. NBC37-1, WP_011979980
Sulfurimonas autotrophica, WP_013326534
Sulfurimonas denitrificans, WP_011371740
Persephonella marina, WP_012675288
Persephonella sp. IF05-L8, WP_029520322
Persephonella sp. KM09-Lau-8, WP_ 029523098
Clade I
Clade II
78
75
100
100
78
100
97
99
99
100
98
91
79
100
97
98
99
0.2

I. AclB

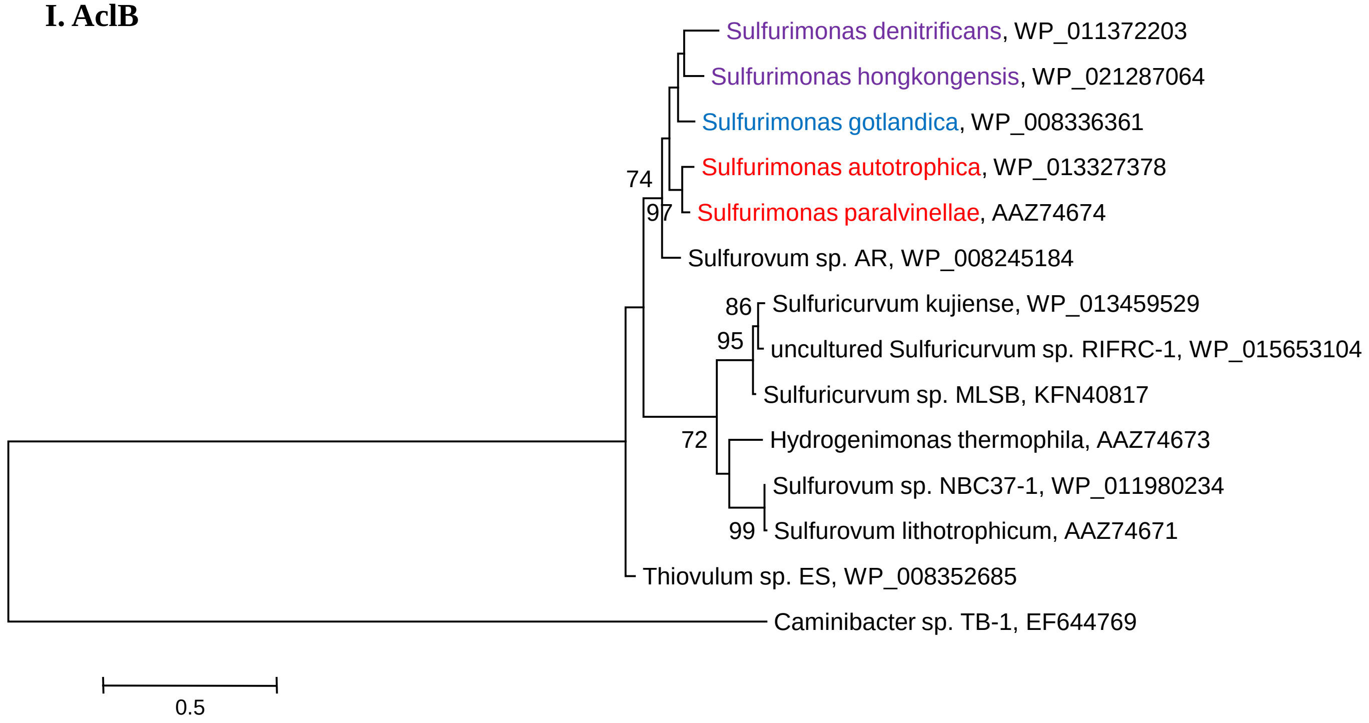

Supplementary Figure S1. Phylogenetic relationships of SoxB (A), SorA (B), ATPS (C), CysH (D), CysI (E), CysC (F), PsrA (G), CcoN (H) and AclB (I) protein sequences from *Sulfurimonas* species.

The SoxB, SorA, ATPS (CysN and Sat-type), CysH, CysI, CysC, PsrA, CcoN and AclB amino acid sequences were compared with the non-redundant protein database of NCBI by using BLASTP. The phylogenetic tree was constructed with MEGA6 (Tamura et al., 2013) based on the Maximum-likelihood method with 1000 bootstrap replications after multiple alignments with ClustalW (Larkin et al., 2007). The percentage of bootstrap resamplings ≥ 70 is indicated on the branches. The scale bar represents the expected number of changes per amino acids position. Isolation sources of *Sulfurimonas* species are indicated in different colors: blue, marine non-vent water system; purple, marine sediments; red, hydrothermal environments.

Reference:

Anderson, I., Sikorski, J., Zeytun, A., Nolan, M., Lapidus, A., Lucas, S., et al. (2011). Complete genome sequence of *Nitratifractor salsuginis* type strain (E9I37-1^T). *Stand. Genomic Sci.* 4**,** 322-330.

Baar, C., Eppinger, M., Raddatz, G., Simon, J., Lanz, C., Klimmek, O., et al. (2003). Complete genome sequence and analysis of *Wolinella succinogenes*. *Proc. Natl. Acad. Sci. USA* 100**,** 11690-11695.

Cai, L., Shao, M.-F., and Zhang, T. (2014). Non-contiguous finished genome sequence and description of *Sulfurimonas hongkongensis* sp. nov., a strictly anaerobic denitrifying, hydrogen- and sulfur-oxidizing chemolithoautotroph isolated from marine sediment. *Stand. Genomic Sci.* 9**,** 1302-1310.

Campbell, B.J., Smith, J.L., Hanson, T.E., Klotz, M.G., Stein, L.Y., Lee, C.K., et al. (2009). Adaptations to submarine hydrothermal environments exemplified by the genome of *Nautilia profundicola*. *PLoS Genet* 5**,** e1000362.

Giovannelli, D., Ferriera, S., Johnson, J., Kravitz, S., Pérez-Rodríguez, I., Ricci, J., et al. (2011). Draft genome sequence of *Caminibacter mediatlanticus* strain TB-2^T, an epsilonproteobacterium isolated from a deep-sea hydrothermal vent. *Stand. Genomic Sci.* 5**,** 135-143.

Grote, J., Schott, T., Bruckner, C.G., Glockner, F.O., Jost, G., Teeling, H., et al. (2012). Genome and physiology of a model Epsilonproteobacterium responsible for sulfide detoxification in marine oxygen depletion zones. *Proc. Natl. Acad. Sci. USA* 109**,** 506-510.

Hamilton, T.L., Jones, D.S., Schaperdoth, I., and Macalady, J.L. (2014). Metagenomic insights into S(0) precipitation in a terrestrial subsurface lithoautotrophic ecosystem. *Front. Microbiol.* 5**,** 756.

Han, C., Kotsyurbenko, O., Chertkov, O., Held, B., Lapidus, A., Nolan, M., et al. (2012). Complete genome sequence of the sulfur compounds oxidizing chemolithoautotroph *Sulfuricurvum kujiense* type strain (YK-1^T). *Stand. Genomic Sci.* 6**,** 94-103.

Larkin, M.A., Blackshields, G., Brown, N.P., Chenna, R., Mcgettigan, P.A., Mcwilliam, H., et al. (2007). Clustal W and Clustal X version 2.0. *Bioinformatics* 23**,** 2947-2948.

Lucas, S., Han, J., Lapidus, A., Cheng, J.-F., Goodwin, L., Pitluck, S., et al. (2015). Complete sequence of *Sulfurospirillum barnesii* SES-3. NCBI database.

Marshall, I.P.G., Blainey, P.C., Spormann, A.M., and Quake, S.R. (2012). A single-cell genome for *Thiovulum* sp. *Appl. Environ. Microbiol.* 78**,** 8555-8563.

Meyer, J.L., and Huber, J.A. (2014). Strain-level genomic variation in natural populations of *Lebetimonas* from an erupting deep-sea volcano. *ISME J.* 8**,** 867-880.

Miller, W.G., Parker, C.T., Rubenfield, M., Mendz, G.L., Wösten, M.M.S.M., Ussery, D.W., et al. (2007). The complete genome sequence and analysis of the Epsilonproteobacterium *Arcobacter butzleri*. *PLoS one* 2**,** e1358.

Nakagawa, S., Takaki, Y., Shimamura, S., Reysenbach, A.L., Takai K, and Horikoshi K (2007). Deep-sea vent ε-proteobacterial genomes provide insights into emergence of pathogens. *Proc. Natl. Acad. Sci. USA* 104**,** 12146-12150.

Park, S.-J., Ghai, R., Martín-Cuadrado, A.-B., Rodríguez-Valera, F., Jung, M.-Y., Kim, J.-G., et al. (2012). Draft genome sequence of the sulfur-oxidizing bacterium "*Candidatus* Sulfurovum sediminum" AR, which belongs to the *Epsilonproteobacteria*. *J. Bacteriol.* 194**,** 4128-4129.

Parkhill, J., Wren, B.W., Mungall, K., Ketley, J.M., Churcher, C., Basham, D., et al. (2000). The genome sequence of the food-borne pathogen *Campylobacter jejuni* reveals hypervariable sequences. *Nature* 403**,** 665-668.

Pati, A., Gronow, S., Lapidus, A., Copeland, A., Glavina Del Rio, T., Nolan, M., et al. (2010). Complete genome sequence of *Arcobacter nitrofigilis* type strain (CI^T). *Stand. Genomic Sci.* 2**,** 300-308.

Sievert, S.M., Scott, K.M., Klotz, M.G., Chain, P.S.G., Hauser, L.J., Hemp, J., et al. (2008). Genome of the epsilonproteobacterial chemolithoautotroph *Sulfurimonas denitrificans*. *Appl. Environ. Microbiol.* 74**,** 1145-1156.

Sikorski, J., Lapidus, A., Copeland, A., Glavina Del Rio, T., Nolan, M., Lucas, S., et al. (2010a). Complete genome sequence of *Sulfurospirillum deleyianum* type strain (5175^T). *Stand. Genomic Sci.* 2**,** 149-157.

Sikorski, J., Munk, C., Lapidus, A., Ngatchou Djao, O.D., Lucas, S., Glavina Del Rio, T., et al. (2010b). Complete genome sequence of *Sulfurimonas autotrophica* type strain ($OK10^T$). *Stand. Genomic Sci.* 3, 194-202.

Takai, K., Campbell, B.J., Cary, S.C., Suzuki, M., Oida, H., Nunoura, T., et al. (2005). Enzymatic and genetic characterization of carbon and energy metabolisms by deep-sea hydrothermal chemolithoautotrophic isolates of *Epsilonproteobacteria*. *Appl. Environ. Microbiol.* 71**,** 7310-7320.

Tamura, K., Stecher, G., Peterson, D., Filipski, A., and Kumar, S. (2013). MEGA6: molecular evolutionary genetics analysis version 6.0. *Mol. Biol. Evol.* 30**,** 2725-2729.

Tan, B., and Foght, J. (2014). Draft genome sequences of *Campylobacterales* (*Epsilonproteobacteria*) obtained from methanogenic oil sands tailings pond metagenomes. *Genome Announc.* 2**,** e01034-01014.

Tomb, J.-F., White, O., Kerlavage, A.R., Clayton, R.A., Sutton, G.G., Fleischmann, R.D., et al. (1997). The complete genome sequence of the gastric pathogen *Helicobacter pylori*. *Nature* 388**,** 539-547.
